# Integrative analyses of transcriptomics and metabolomics upon seed germination of foxtail millet in response to salinity

**DOI:** 10.1038/s41598-020-70520-1

**Published:** 2020-08-12

**Authors:** Jiaowen Pan, Zhen Li, Shaojun Dai, Hanfeng Ding, Qingguo Wang, Xiaobo Li, Guohua Ding, Pengfei Wang, Yanan Guan, Wei Liu

**Affiliations:** 1 Biotechnology Research Center, Key Laboratory of Genetic Improvement, Ecology and Physiology of Crops, Shandong Academy of Agricultural Sciences, Jinan, 250100 Shandong People’s Republic of China; 2 Crop Research Institute, Shandong Engineering Laboratory for Featured Crops, Shandong Academy of Agricultural Sciences, Jinan, 250100 Shandong People’s Republic of China; 3 Shandong Center of Crop Germplasm Resources, Shandong Academy of Agricultural Sciences, Jinan, 250100 Shandong People’s Republic of China; 4 College of Life Sciences, Shandong Normal University, Jinan, 250014 Shandong People’s Republic of China; 5 School of Life Science and Technology, Harbin Normal University, Harbin, 150025 Heilongjiang People’s Republic of China; 6 Development Center of Plant Germplasm Resources, College of Life Sciences, Shanghai Normal University, Shanghai, 200234 People’s Republic of China; 7 Shandong Engineering Research Center for Grape Cultivation and Deep-Processing, Shandong Academy of Grape, Jinan, 250100 Shandong People’s Republic of China

**Keywords:** Physiology, Plant sciences

## Abstract

Salinity stress has become an expanding threat to food security worldwide. Revealing the mechanisms of salinity tolerance in plants has immense significance. Foxtail millet (*Setaria italica* L.) has been regarded as a model crop for exploring mechanisms under stress, considering its extreme adaptation abilities to adverse ecologies. In present study, two foxtail millet cultivars of Yugu2 and An04 with contrasting salt tolerance properties were investigated through integrative analyses of transcriptomics and metabolomics. In the transcriptomics results, 8887 and 12,249 DEGs were identified in Yugu2 and An04 in response to salinity, respectively, and 3149 of which were overlapped between two varieties. These salinity-responsive genes indicated that ion transport, redox homeostasis, phytohormone metabolism, signaling and secondary metabolism were enriched in Yugu2 by GO and KEGG analyses. The integrative omics analysis implied that phenylpropanoid, flavonoid and lignin biosynthesis pathways, and lysophospholipids were vital in determining the foxtail millet salinity tolerance. Importantly, the tolerance of Yugu2 attributed to higher efficiencies of ion channel and antioxidant system. All these provide a comprehensive regulatory network of foxtail millet to cope with salinity, and shed some lights on salt tolerance which is relevant for other cereal crops.

## Introduction

Soil salinity is a growing problem for irrigated agriculture, and it severely limits the productivity and geographical distribution of crops. About 800 × 10^6^ million ha of land is affected by salinity with an annual increase of ∼1–2% worldwide^1^. High salinity first leads to osmotic stress and then ion toxicity to plants, with detrimental effects in inhibiting all the major physiological processes such as germination, growth and morphogenesis, photosynthesis, nutrient absorption, and yield of plants^2^.

To mount an effective response to cope with salinity, plants have evolved abilities to sense and manage both the osmotic stress and ions toxicity. The sensory modality of plants in response to NaCl was distinct from the response to purely osmotic stress^3^. However, it is not completely understood whether plants have receptor or sensor for Na^+4^. As per reported, Na^+^ enter cytoplasm probably via various cation exchange channels, such as nonselective cation channel (NSCC), high-affinity K^+^ channel (HKT), the low affinity K^+^ channel^4^. The salt stress also results in rapid increase in cytosolic Ca^2+^ and initiates Ca^2+^ signal propagation, and then activates Na^+^ efflux pathway^5^. The calcium binding proteins such as SOS3 and SOS3-Like calcium binding protein 8 (SCaBP8) could perceive calcium signal. The Na^+^/H^+^ exchanger of SOS1 could be activated by SOS3/SOS2 complex to exclude Na^+^ from the cytosol into the apoplast^4,6^, and it also could be activated by mitogen-activated protein kinase 6 (MPK6) in phosphatidic acid (PA)-mediated salt-stress signaling pathway^7^.

In secondary injury responses of salt stress, synthesis and accumulation of compatible osmolytes are fundamental adaptive strategy to lower the cell osmotic potential and stabilize proteins and cellular structures. The representative osmolytes have been identified in many species, such as proline, glycine betaine, sugars, polyols including mannitol, glycerol, and sorbitol^3^. Recently, the strategies of high-throughput omics, such as transcript profiling, proteomics and metabolomics, have been developed, which are pivotal to reveal the regulatory networks and metabolic rearrangements in controlling plant salinity tolerance^4^.

Foxtail millet (*Setaria italic* L.) is a particularly important food and fodder cereal crop, and remarkably adaptable to adverse ecologies, including drought, extreme temperature, and high soil salinity^8^. It possesses attractive qualities, such as small diploid genome (~ 510 Mb), lower repetitive DNA, short life cycle, and C_4_ photosynthesis. These characteristics promote it as a model crop for exploring basic biology processes, such as plant architecture, physiology and genome evolution^9,10^. The drought responses molecular mechanisms of foxtail millet have been investigated and the genome-wide drought responsive genes, proteins and miRNAs have been identified^9–11^. However, the salinity response mechanisms of foxtail millet have not been well characterized.

In order to create the regulatory network of salinity response, the salt-tolerance of different foxtail millet varieties were screened. Based on the phenotypic alteration and physiological indexes determination under 150 mM NaCl treatment, Yugu2 was defined as salt tolerant variety and An04 was identified as salt sensitive. Integrative analyses of transcriptomics and metabolomics demonstrated that several key biological processes and metabolites, such as ion transport, redox homeostasis, secondary metabolism and lysophospholipids, were vital for Yugu2 salt tolerance. The data derived here may facilitate to establish of salt responsive network of foxtail millet, and provide clues and guidelines for salt tolerant germplasm cultivation and innovation of cereal crops.

## Results

### Screening of foxtail millet salt-tolerance varieties

**Figure 1:**
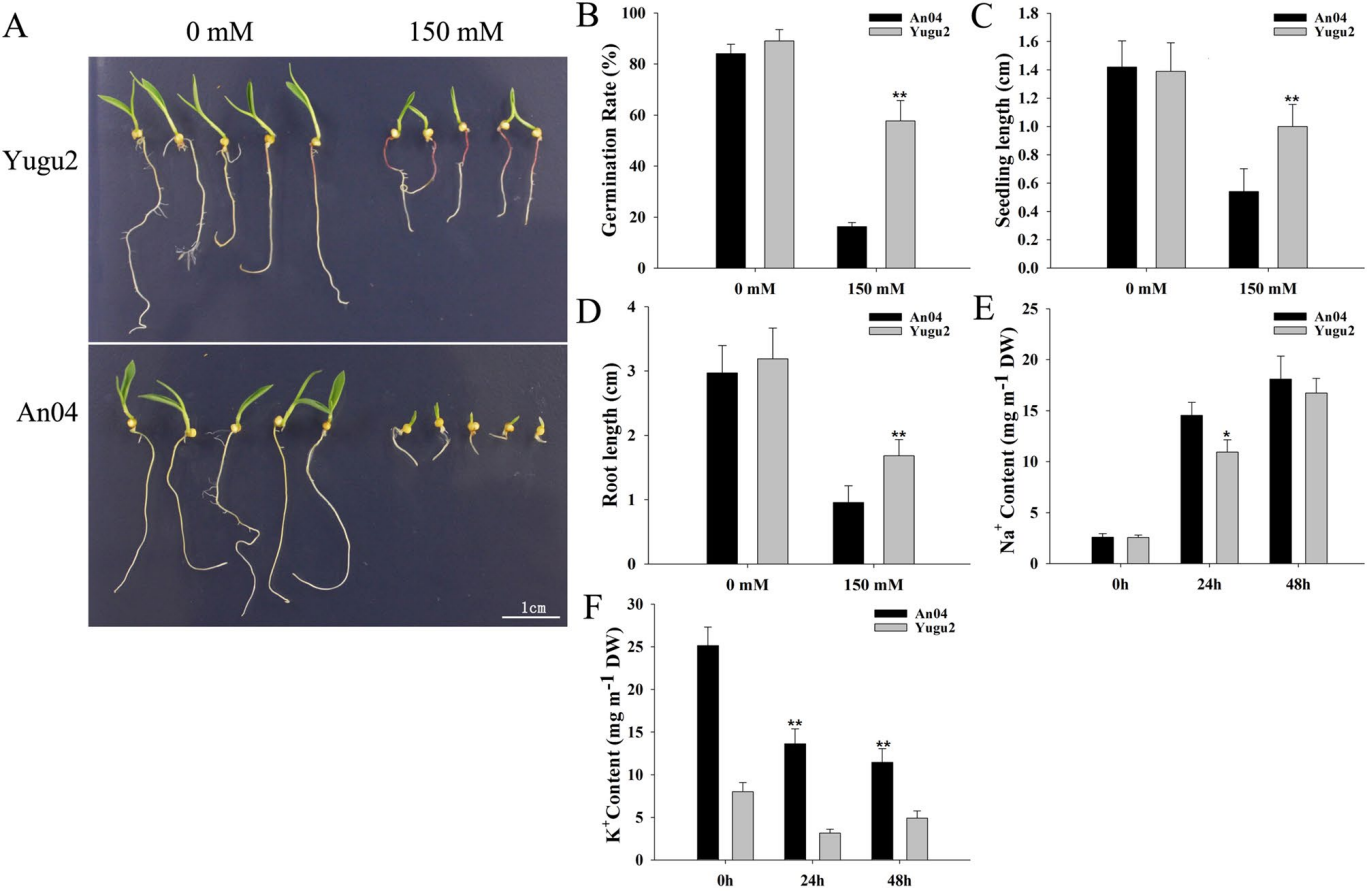
Effects of salinity on the two foxtail millet varieties. (**A)** The phenotypes of foxtail millet seeds germinated under salt stress for 7 days. (**B**–**D**) The germination rate, seedling and root length of Yugu2 and An04 after germinated for 7 days under salinity. (**E**, **F**) The Na^+^ and K^+^ contents in the roots of Yugu2 and An04 under salinity. Error bars indicate ± SE of at least three biological repeats. Student’s t-test was carried out to analyze significant differences between Yugu2 and An04, **indicates the *p* value < 0.01 and *indicates the *p* value < 0.05, respectively. The SigmaPlot Version 11.0 was used to draw histograms in (**B**–**F**).

Fourteen Foxtail millet varieties at germination stage were used for salt-tolerance screening. The germination rate, seedling length, and root length of 7-day old seedlings were all significantly inhibited under salinity (Fig. S1), and the inhibition of An04 was more obviously than that of Yugu2 (Fig. 1A–D and Fig. S1). The roots of Yugu2 appeared purple-red (Fig. 1A). The Na^+^ contents increased dramatically in An04 than that of Yugu2 under salinity (Fig. 1E), while K^+^ contents in An04 and Yugu2 were all decreased (Fig. 1F), indicating the disruption of ion balance in both varieties. Therefore, An04 and Yugu2 were defined as salt sensitive variety and salt tolerance variety, respectively, which were selected for salt response investigation.

### Differentially expressed genes (DEGs) between Yugu2 and An04 under salinity

The RNA-Seq was performed using the root samples of An04 and Yugu2 seedlings, which were named as An04C or YG2C for untreated control, and An04T or YG2T for salt treatment, respectively. Considering three independent biological replicates for each sample, there were twelve libraries were constructed and sequenced, and totally of approximately 626 million raw reads were generated. After removing adapter sequence and low-quality data, a total of 597 million (95.39%) clean reads, which representing an average of 49.8 million clean reads per sample were obtained (Table 1). Approximately over 86% reads were mapped to the *Setaria italic* reference genome, and over 84% reads were mapped to uniquely regions (Table 1). The FPKM (expected number of Fragments Per Kilobase of transcript sequence per Millions base pairs sequenced) density distribution (Fig. S2A) and FPKM violin diagram (Fig. S2B) suggested that the detected genes followed a standard normal distribution, which fully proved the high quality and reasonable reproducibility of the sequenced data.

**Table 1:** Quality statistics of read numbers of foxtail millet transcriptomics.

Sample name	Raw reads	Clean reads	Clean bases	Error rate (%)	Q20 (%)	Q30 (%)	GC content (%)	Total mapped	Multiple mapped	Uniquely mapped
YG2C1	53,517,332	50,631,174	7.59G	0.03	97.50	93.55	60.85	44,815,831 (88.51%)	574,282 (1.13%)	44,241,549 (87.38%)
YG2C2	53,333,334	51,643,874	7.75G	0.03	95.23	88.79	59.17	45,003,025 (87.14%)	597,010 (1.16%)	44,406,015 (85.99%)
YG2C3	53,333,334	51,618,960	7.74G	0.03	95.77	89.80	59.18	45,510,138 (88.17%)	594,516 (1.15%)	44,915,622 (87.01%)
YG2T1	49,353,206	46,959,552	7.04G	0.03	97.13	92.87	62.48	40,683,883 (86.64%)	625,995 (1.33%)	40,057,888 (85.3%)
YG2T2	46,628,886	44,137,084	6.62G	0.03	96.40	91.27	62.18	38,042,346 (86.19%)	568,696 (1.29%)	37,473,650 (84.9%)
YG2T3	49,504,784	47,304,220	7.1G	0.03	96.14	90.81	61.98	40,710,673 (86.06%)	629,182 (1.33%)	40,081,491 (84.73%)
An04C1	53,333,334	50,961,024	7.64G	0.03	96.11	90.76	59.30	45,945,652 (90.16%)	596,842 (1.17%)	45,348,810 (88.99%)
An04C2	53,333,334	51,068,190	7.66G	0.03	96.00	90.51	59.17	45,994,169 (90.06%)	603,059 (1.18%)	45,391,110 (88.88%)
An04C3	53,333,334	50,982,896	7.65G	0.03	95.49	89.42	58.58	45,716,638 (89.67%)	613,002 (1.2%)	45,103,636 (88.47%)
An04T1	53,333,334	50,723,764	7.61G	0.03	97.17	92.87	60.09	44,572,801 (87.87%)	721,076 (1.42%)	43,851,725 (86.45%)
An04T2	53,333,334	50,518,270	7.58G	0.03	97.47	93.46	60.52	44,541,380 (88.17%)	716,572 (1.42%)	43,824,808 (86.75%)
An04T3	54,235,694	51,145,870	7.67G	0.03	97.49	93.61	61.92	44,708,640 (87.41%)	689,511 (1.35%)	44,019,129 (86.07%)

Total Raw Reads: The number of reads before filtering; Total Clean Reads: Filtered reads; Total Clean Bases (Gb): Total number of bases after filtration; Q20 (%): Proportion of nucleotides with a quality value larger than 20 in the filtered reads; Q30 (%): Proportion of nucleotides with a quality value larger than 30 in the filtered reads.

**Figure 2:**
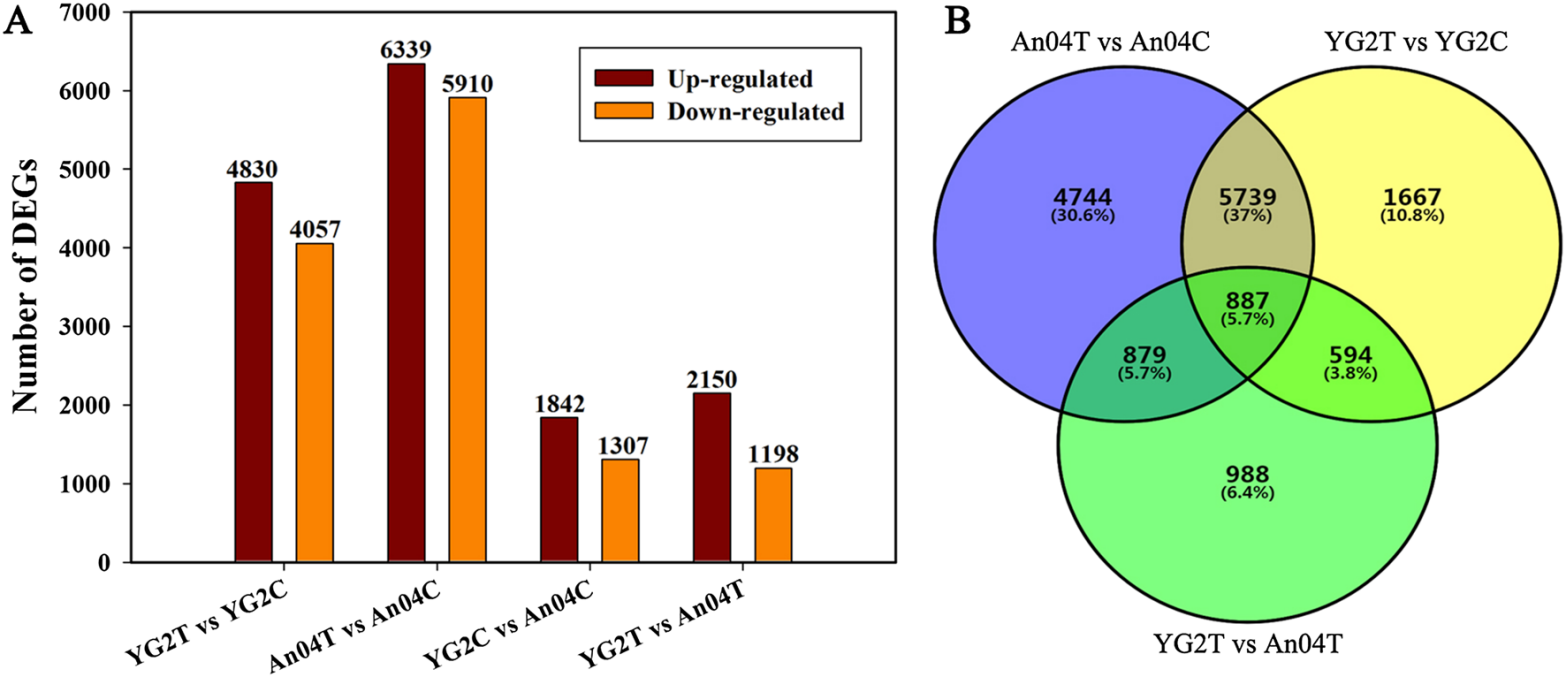
Gene expression analyses of Yugu2 and An04 subjected to salt stress. (**A**) The numbers of DEGs in different comparison groups. (**B**) Venn diagrams exhibiting the overlap of DEGs among different comparisons. The SigmaPlot Version 11.0 was used to draw histograms in (**A**). VENNY 2.1 (https://bioinfogp.cnb.csic.es/tools/venny/) was used to create the (**B**).

DGEs were identified based on the criteria of absolute log2 Fold Change ≥ 1 and q-value < 0.05. A total of 8887 and 12,249 DEGs were identified in salt-tolerance (YG2TvsYG2C) and salt-sensitive (An04TvsAn04C) variety, respectively (Fig. 2A and Table S1). Among which 4830 and 6339 genes were up-regulated, and 4057 and 5910 genes were down-regulated in Yugu2 and An04 under salinity, respectively (Fig. 2A and Table S1). There were 3149 DEGs and 3348 DEGs identified in YG2CvsAn04C and YG2TvsAn04T (Fig. 2A and Table S1). The intersection of YG2TvsYG2C with An04TvsAn04C , which represent the amount of DEGs in Yugu2 and An04 after been treated by salt, was 6626, among which only 887 DEGs were shared in these two varieties under salinity (intersecting with YG2TvsAn04T) (Fig. 2B). There were 1481 DEGs were obtained from the intersection of YG2TvsYG2C with YG2TvsAn04T, which represent the dramatically salt responsive genes in Yugu2.

### Functional categorization of DEGs

The DEGs were mainly enriched in the processes of metabolic, cellular and biological regulation of GO sub-categories of biological process (GOBP) (Fig. S3A). Catalytic activity, binding and transporter activity were the top GO sub-categories of molecular function (GOMF), while the GO sub-categories of cellular component (GOCC) were mainly focused in cell and membrane (Fig. S3A). The up-regulated DEGs of Yugu2 were mainly included trans-membrane transport (GO:0055085), oxidation–reduction process (GO:0055114), response to oxidative stress (GO:0006979) and cellular potassium ion transport (GO:0071804) (Table S2), while up-regulated DEGs of An04 were significantly enriched of protein phosphorylation (GO:0006468), cellular protein modification process (GO:0006464) (Table S3). For the down-regulated DEGs, the enriched terms mainly encode for metabolic and biosynthetic processes, such as macromolecule biosynthetic process, amide biosynthetic process, and peptide metabolic and biosynthetic process (Tables S2 and S3). These repressed biosynthetic processes implied that the cell metabolisms are incline to inhibit root growth of foxtail millet under salinity stress (Fig. 1).

The most enriched GOBP of YG2CvsAn04C and YG2TvsAn04T were metabolic and cellular process, and their GOMF mainly focused on catalytic activity, binding, transporter activity, and antioxidant activity, and GOCC especially related to cell, organelle and membrane (Fig. S3). In the up-regulated DEGs of YG2CvsAn04C, the mainly enriched sub-categories were mainly involved in protein modification (GO:0036211), metabolic process (GO:0008152) and oxidation–reduction process (GO:0055114). For the YG2TvsAn04T up-regulated DEGs, the enriched sub-categories with a large number of genes included oxidation–reduction process (GO:0055114), protein phosphorylation (GO:0006468), signal transduction (GO:0007165), trans-membrane transport (GO:0055085), response to oxidative stress (GO:0006979), and nuclear ubiquitin ligase complex (GO:0000152) (Tables S4 and S5).

**Figure 3:**
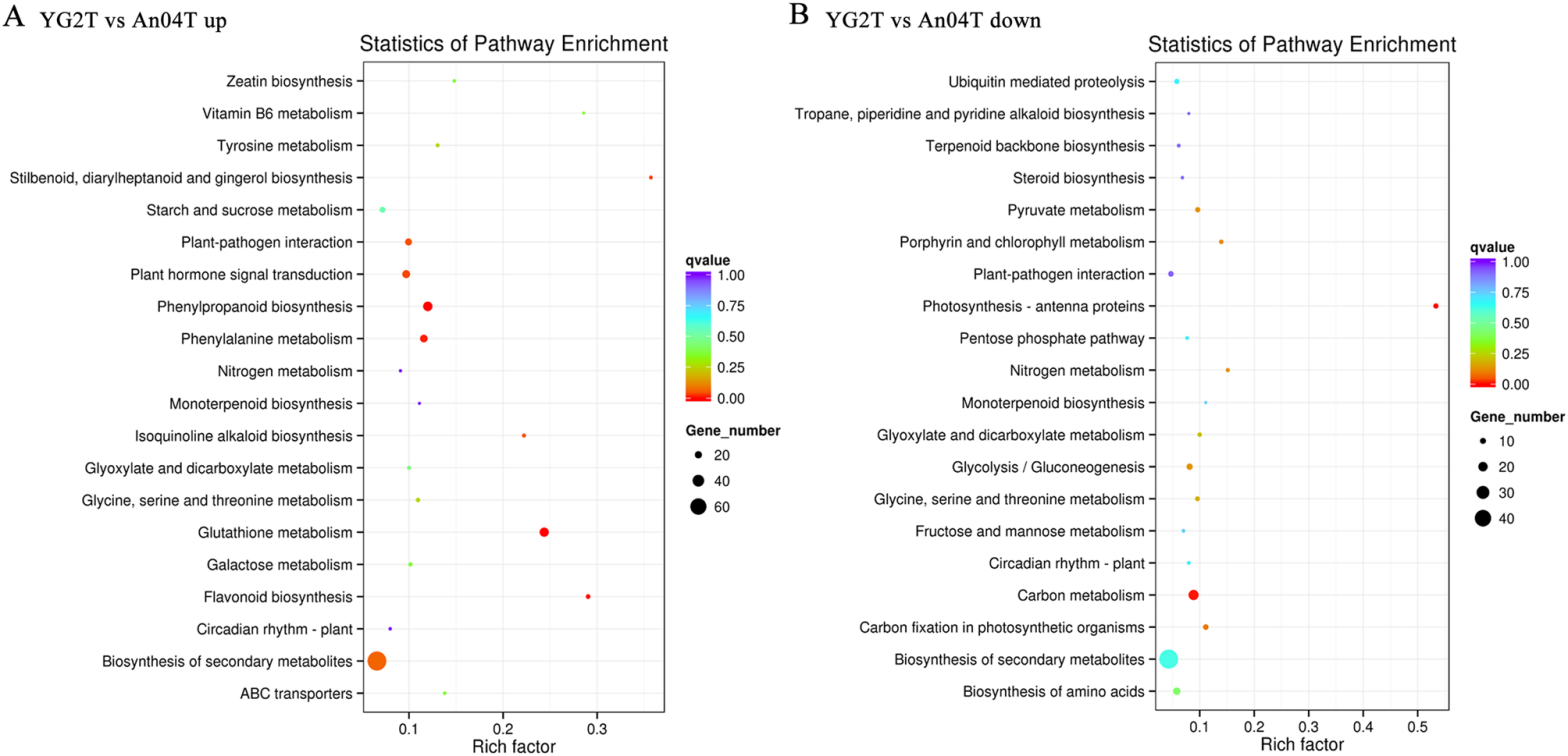
Scatter plot of top 20 KEGG pathways for the up-regulated genes (**A**) and down-regulated genes (**B**) in YG2TvsAn04T. The abscissa means rich factor reflecting the proportion of DEGs in a given pathway. The circle area indicated the number of DEGs and the circle color represented the ranges of the corrected *P* value.

KEGG pathway enrichment analysis within and between Yugu2 and An04 under salinity showed that phenylpropanoid biosynthesis, phenylalanine metabolism, biosynthesis of secondary metabolites, and glutathione metabolism pathways were all significantly enriched in up-regulated genes (Fig. 3, Figs. S4 and S5, Tables S6–S9). In addition, the plant hormone signal transduction pathway also enriched in YG2TvsAn04T (Fig. 3, Table S9). However, pathways related to biomass accumulation such as starch and sucrose metabolism, fatty acid metabolism and metabolic pathways were inhibited in An04TvsAn04C and YG2TvsYG2C (Fig. S4, Tables S6 and S7).

### K-means clustering and functional enrichment of DEGs

**Figure 4:**
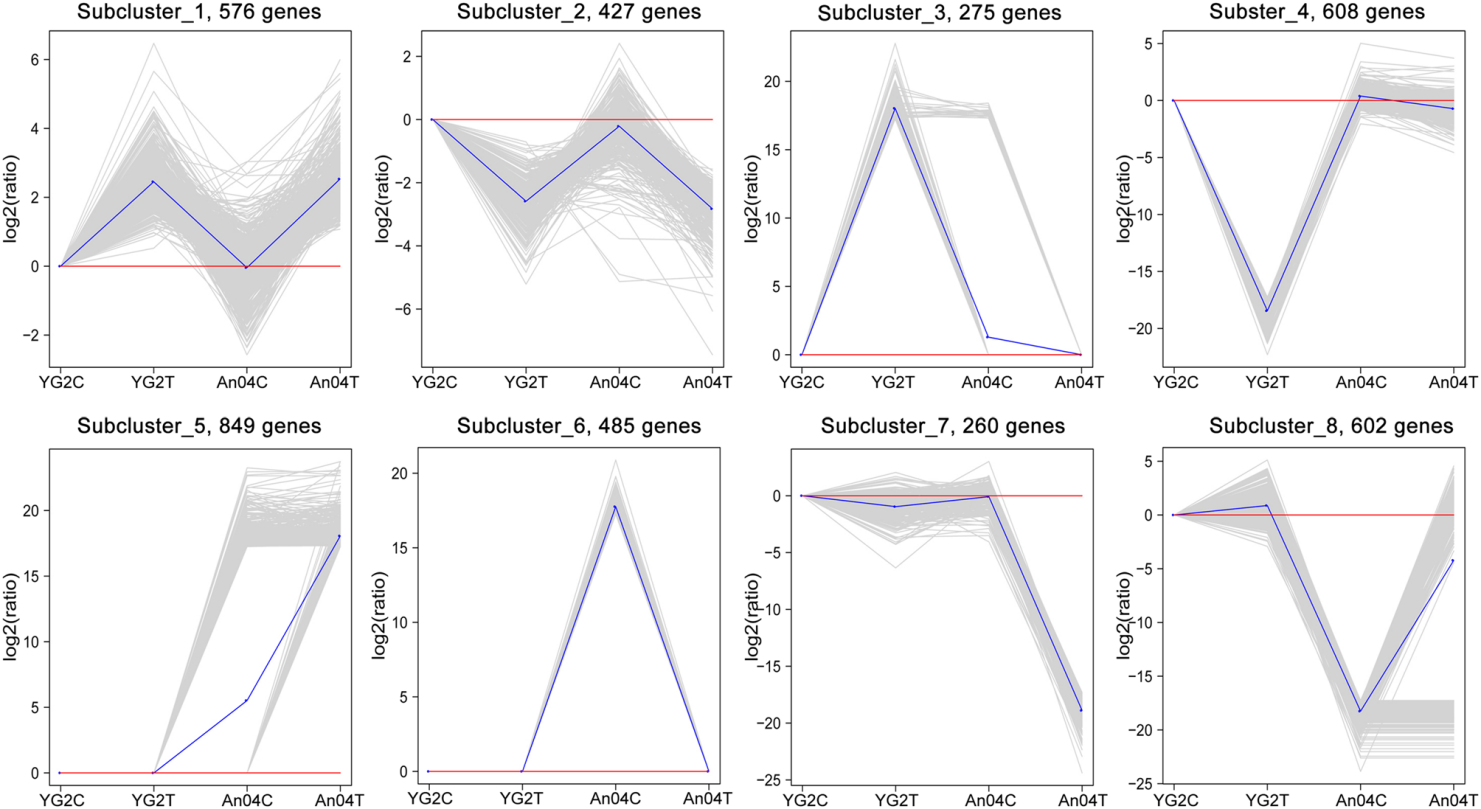
K-means clustering of gene expression profiles in Yugu2 and An04 during salt stress. The x-axis indicates the samples, and y-axis represents the relative expression level. Gray lines exhibit the relative expression levels of DEGs in different samples before and after treatment, blue line represents the average values of relative expression in each sub-cluster. NbClust package of R (V 1.4 https://cran.r-project.org/web/packages/NbClust/index.html) was used to create this figure.

To investigate the expression patterns of DEGs under salinity more intuitively, K-means clustering analysis was adopted (Fig. 4 and Table S10). There were 576 up-regulated and 427 down-regulated DEGs in both cultivars, which were classified into sub-cluster 1 and sub-cluster 2, respectively. DEGs of sub-cluster 1 mainly enriched of metabolic process, oxidation–reduction process, and organic substance biosynthesis. DEGs of sub-cluster 2 mainly enriched of regulation of metabolic process and nitrogen compound metabolic process. Partial DEGs in Yugu2 could mainly be grouped into sub-cluster 3 and sub-cluster 4. Sub-cluster 3 included 275 salt induced genes that participate in response to stimulus, oxidation–reduction process and primary metabolic process. The sub-cluster 4 contained 608 salinity-reduced genes that belong to various pathways (Fig. 4). Considering the salt tolerant property of Yugu2, the DEGs displayed in sub-cluster 3 may be critical during salinity response process. Sub-cluster 5 and 6 contained 849 and 458 salt up-regulated genes in An04, respectively. And these DEGs mainly participated in cellular metabolic process, organic substance metabolic process, and protein metabolic process. The higher basal metabolic levels of An04 displayed in the sub-cluster 5 and 6 compared with those of Yugu2 were consistent with previous reported that An04 produced significant higher crop yields under well-watered conditions^9^. Sub-cluster 7 and 8 included 260 and 602 salinity inhibited genes in An04, respectively. The DEGs in sub-cluster 7 mainly involved in carbohydrate metabolic process, cytoskeleton organization and oxidation–reduction process. The DEGs in sub-cluster 7 could also become the better evidence to explain the phenotypes of severe inhibition of root growth of An04 under salinity.

### Key genes responsible for salt tolerance in Yugu2

Considering the salt responsive genes showed differential expressions in the YG2CvsAn04C and YG2TvsAn04T, the DEGs in these two comparisons might be vital for salinity tolerance in Yugu2.

As the key factors for influx of apoplastic Ca^2+^ into the cytosol and expulsion of cytosolic Ca^2+^, cyclic nucleotide-gated ion channel (CNGC) (Si024243m.g), signaling transduction components of calcium-dependent protein kinase (CDPK) and salt overly sensitive (SOS) pathway were all up-regulated in YG2TvsAn04T (Table S11). DEGs of 9-cis-epoxycarotenoid dioxygenase 1 (NCED1) (Si024703m.g) that participated in ABA biosynthesis and ABA receptor PYL, protein phosphatase 2C, and kinases SnRK2, that act as central components in ABA signal transduction, were also increased in YG2TvsYG2C and An04TvsAn04C. In view of important role of mitogen-activated protein kinase (MAPK) cascades in salinity response in plants, the components of MAPK cascades were also investigated. Four genes encoding MAP kinase kinase kinase (MAPKKK/MEKK) and two genes encoding MAP kinase kinase (MAPKK/MKK) were up-regulated in YG2TvsYG2C and An04TvsAn04C except six genes encoding MAP kinase (MAPK/MPK) were down-regulated (Table S11).

Sixty-nine types of transcription factors (TFs) were identified in our transcriptomes. As the concerned key regulators under salinity, the induction of five MYB, eight bHLH, seven dehydration-responsive element-binding proteins (DREB), and ten ethylene-responsive factors (ERFs) were detected in YG2TvsAn04T and YG2TvsYG2C under salinity (Table S11).

DEGs related to transporters and H^+^-ATPase, which could act coordinately to maintain cellular ion homeostasis, also have been identified^4^. The orthologs of SOS2 and SOS3 in foxtail millet, such as Si035799m.g, Si029714m.g, Si026897m.g and Si011148m.g, all showed significantly higher expression levels in Yugu2 than those in An04 before and after salt stress (Table S11). Meanwhile, AtAVP1 homologous of AVP1 (Si000395m.g) that may mediate vacuolar Na^+^ sequestration in foxtail increased about 2.8-fold in YG2TvsAn04T. Two orthologs of high-affinity K^+^ channel (HKT) (Si001234m.g and Si001214m.g) that participate in distribution of Na^+^ between root and shoot to prevent it over-accumulated were found to express high in Yugu2, but the variation quantities and trends of K^+^ channels all were less than those of AKT1, HAK, and KOR in An04 (Table S11). The results of higher activities of K^+^ channels indicated that more K^+^ were required and flowed into cells of An04 to maintain the ion homeostasis under salt stress.

**Figure 5:**
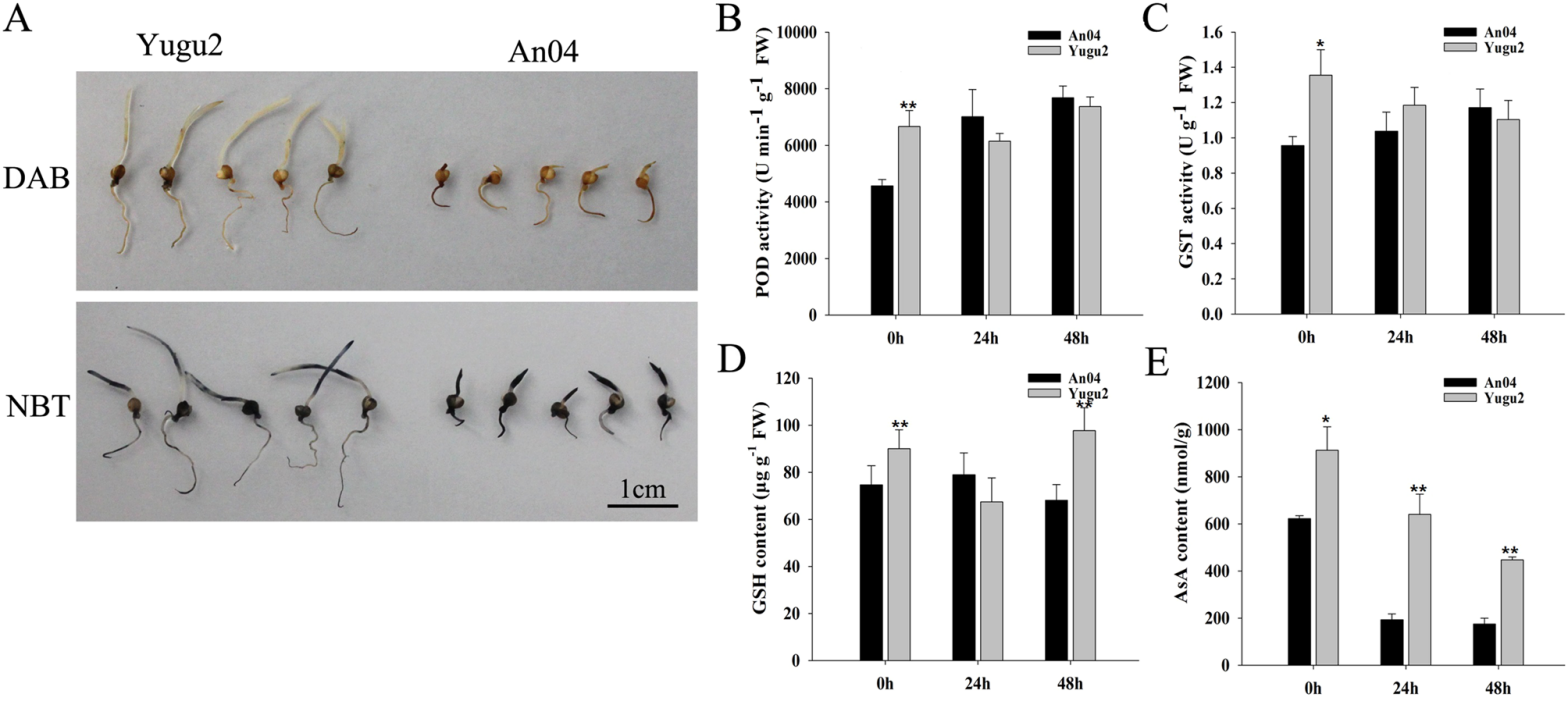
ROS accumulation, activity of antioxidant enzymes, and contents of GSH and AsA in Yugu2 and An04 under salinity. (**A**) In situ detection of H_2_O_2_ and O_2_^−^ by DAB and NBT staining of 7-day germinated seedlings under salt stress. 7-day-old hydroponic seedlings were treated with 150 mM NaCl for 0 h, 24 h and 48 h, and the roots of each samples were sampled for later use. The activities of the POD (**B**) and GST (**C**), and the contents of GSH (**D**) and AsA (**E**) were measured. Error bars indicate ± SE of at least three biological repeats. Student’s t-test was carried out to analyze significant differences between two varieties, **indicates the *p* value < 0.01 and *indicates the *p* value < 0.05, respectively. The SigmaPlot Version 11.0 was used to draw histograms in **B**–**E**.

Salinity-induced ROS injury must be tightly regulated in plant cells^4^. Although ascorbate (AsA)–glutathione (GSH) cycle related four ascorbate peroxidase (APX) and one monodehydroascorbate reductase (MDAR) genes were down-regulated in both varieties (Table S11). There were 32 glutathione transferase (GST) and one glutathione peroxidase (GPX) genes were up-regulated under salinity, and all of the genes expressed higher in Yugu2 than those in An04. Eight peroxidase (POD) genes were up-regulated in YG2TvsAn04T (Table S11). Further DAB (3,3′-diaminobenzidine) and NBT (nitrobluetetrazolium) staining also showed that a greater accumulation of H_2_O_2_ and O_2_^-^ in An04 than that in Yugu2 (Fig. 5A). As the consequences of higher expression levels of POD and GST in Yugu2 (Fig. 5B and C), the contents of glutathione in Yugu2 were 20% and 43% above that in the An04 after been treated for 0 h and 48 h, respectively (Fig. 5D). The AsA contents were about 1.5, 3 and 2.5-fold higher in Yugu2 than in An04 at 0 h, 24 h and 48 h, respectively (Fig. 5E). Those results were consistent with the changes of ROS related DEGs, and that the Yugu2 had a relatively efficient antioxidant system in maintaining the ROS homeostasis under stress.

Salt stress greatly suppresses root meristem growth by inhibiting PINFORMED (PIN) genes expression, which in turn reduce the auxin levels^12^. In our study, the expressions of five PIN genes were significantly repressed in An04, while the suppression was more alleviated in Yugu2. Consistent with the changes of the PIN genes, the expressions of auxin-responsive proteins of *IAA* were also inhibited in both varieties, but there were five *IAA* genes had increased expressions in YG2TvsAn04T. In addition, three auxin biosynthetic related genes of indole-3-pyruvate monooxygenase (YUCCA) (Si003703m.g, Si003842m.g and Si012015m.g) were inhibited in Yugu2 and An04 under salinity, but two *YUCCA* genes (Si001725m.g and Si040872m.g) were up-regulated in YG2TvsAn04T (Table S11). The GA biosynthesis and signaling related genes were also identified. GA20-oxidases and GA2-oxidases were the key enzymes involved in the formation of the bioactive GA and deactivation of GA, respectively. One GA20-oxidase gene was down-regulated, and two GA2-oxidases were up-regulated in Yugu2 and An04 after salt stress (Table S11). Two DELLA genes, which were critical regulators of GA signaling, also increased in YG2TvsAn04T.

Glycerol-3-phosphate acyltransferases (GPAT) are a group of important enzymes involved in plant lipid, suberin and cutin biosynthesis, it catalyzes the acylation of glycerol-3-phosphate to produce lysophosphatidic acids^13,14^. Lysophospholipids are found as minor membrane components and signaling mediators, and could be accumulated in response to biotic and abiotic stress. Seven genes encoding GPAT were detected to be up-regulated in salt-treated Yugu2 and An04 (Table S11). Long chain acyl-CoA synthetase (LACS) activated the free fatty acid precursors into fatty acyl-CoA thioesters, and fatty acyl-CoA reductase (FACR) catalyzes acyl group of fatty acyl-CoA to fatty alcohol. These enzymes catalyze critical steps for metabolism of both wax and cutin^15^. Two *LACS* and four *FACR* genes had increased expression levels in both Yugu2 and An04 after salt stress. Eleven cinnamoyl-CoA reductase (*CCR*), seven cinnamyl alcohol dehydrogenase (*CAD*), and 3-ketoacyl-CoA synthase (*KCS*), which encoding the key enzymes involved in the biosynthesis of lignin and very long chain fatty acids (VLCFAs), showed increased expression levels after salt stress (Table S11). Among these up-regulated genes, four *CCR*, two *CAD* and four *KCS* genes had higher expression levels in YG2TvsAn04T. These salt response genes may contribute to synthesis of waxy cuticle, which in turn could limit water loss in membranes to cope with osmotic stress.

A common consequence of plant affected by salt stress is the accumulation of compatible osmolytes^3^. A gene encoding delta-1-pyrroline 5-carboxylase synthetase (P5CS), which catalyze the rate-limiting steps of proline biosynthesis, was significantly up-regulated in Yugu2 and An04. Salt stress induced NADP-dependent malic enzyme (NADP-ME), which could provide NADPH for synthesis of proline^16^, were also identified in up-regulated DEGs of Yugu2 and An04. Amylases catalyzed remobilize starch reserve to release sugars and derived metabolites, which could function as osmoprotectants and contribute to mitigate the stress^17^. Four alpha-amylase and three beta-amylase had increased expression levels in YG2TvsAn04T and YG2TvsYG2C (Table S11). The expressions of four cellulose synthases were obviously up-regulated, and among them, one gene (Si009413m.g) was up-regulated over 11-fold in YG2TvsAn04T. Xyloglucan endotransglucosylase/hydrolase (XTH) is another pivotal enzyme regulating cell wall construction and metabolism, and can relieve the salinity-induced decrease in wall extensibility^18,19^. The expression levels of four *XTH* genes were down-regulated in Yugu2 and An04 after salt stress, but these genes showed increased expression levels in YG2TvsAn04T (Table S11).

### Validation of DEGs using qRT-PCR

**Figure 6:**
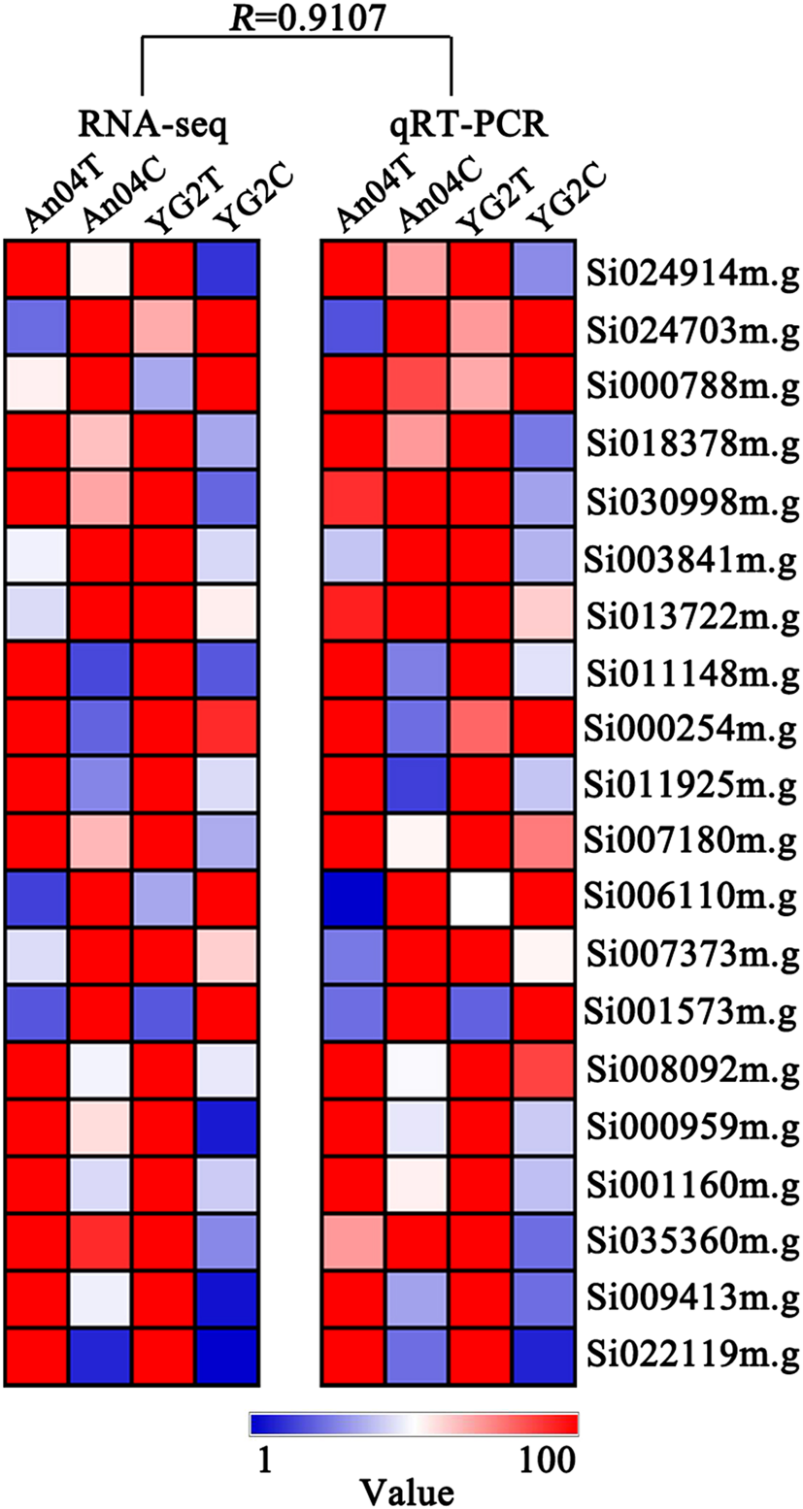
qRT-PCR verification of selected DEGs in Yugu2 and An04 under salt. R indicates the correlation coefficient between RNA-seq data and qRT-PCR.

To verify the RNA-seq data, 20 DEGs which involved in the processes of signal transduction, transcription factor regulation, phytohormone and compatible osmolytes metabolism were randomly selected, and qRT-PCR were performed. The results showed similar expression patterns and high correlation coefficient of 0.9107 between qRT-PCR with RNA-seq (Fig. 6), which demonstrated creditable and repeatable of the data.

### Widely targeted metabolic profiling assay in Yugu2 root

**Figure 7:**
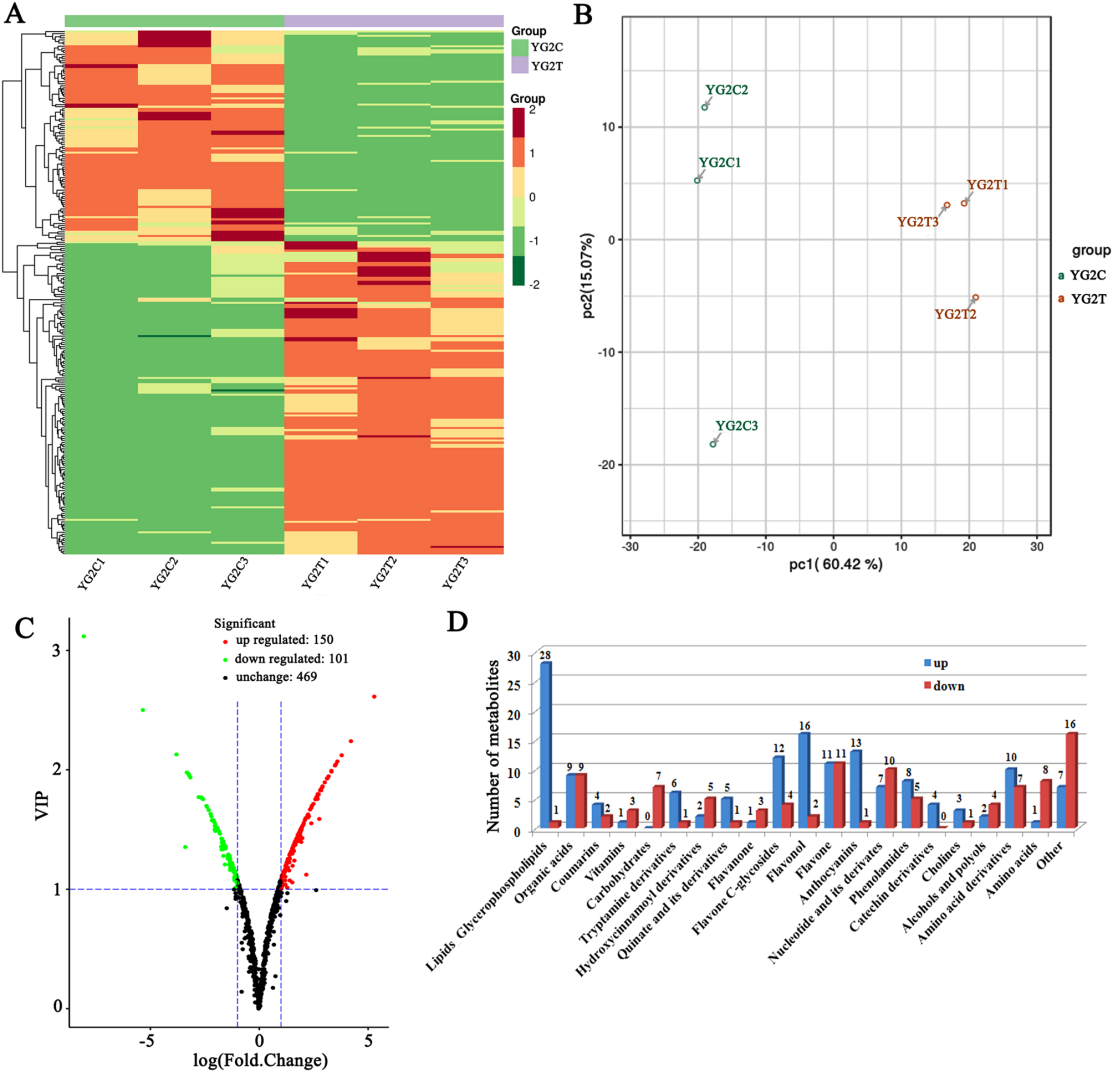
Heat map visualization and PCA of differential metabolites in Yugu2. (**A**) Heat map visualization. The content of each metabolite was normalized to complete linkage hierarchical clustering. Each example indicated in a single column and each metabolite was showed by a single row. Green and red indicates the down-regulated and up-regulated metabolites, respectively. The color key scale indicated the abundance of metabolites. (**B**) Score plots for principle components 1 and 2 exhibited great cohesion within groups and good separation between control and salt treatments. (**C**) The volcano plot indicates the differential metabolites in Yugu2 before and after salt stress. (**D**) The number of differential metabolites in each class. Pheatmap package of R (v1.0.12, https://cran.r-project.org/web/packages/pheatmap/index.html) was used to create the (**A**). Ggplot package of R (v3.2.1, https://cran.r-project.org/web/packages/ggplot2/index.html) was used to create the (**B**). Ropls package of R (v1.18.8, https://bioconductor.org/packages/release/bioc/html/ropls.html)used to create the (**C**). Microsoft Office Excel (2007) used to create the (**D**).

To clarify the metabolic accumulation patterns of foxtail millet under salinity stress, the widely targeted metabolomics assay was performed and Yugu2 roots were used. Totally of 720 metabolites were identified (Fig. 7A and Table S12). Principal component analysis (PCA) have shown that the metabolites mainly grouped into two distinct areas in the plot (Fig. 7B), which revealing the distinct metabolic profiles in the roots of Yugu2 before and after salt treatment. Further analysis showed that the contents of totally 251 metabolites, which mainly concerned with lipids glycerophospholipids, organic acids, carbohydrates, phenolic compounds, amino acid and others, were changed dramatically in coping with salt (Fig. 7C and Table S13). Therein the contents of 150 metabolites were increased, and remaining were decreased (Fig. 7C and Table S13). Among up-regulated metabolites, the contents of flavonoids, including anthocyanins, flavones, flavonols, and flavanones were significantly accumulated (Fig. 7D and Table S13). Especially the contents of cyanidin 3-O-rutinoside, cyanidin 3-O-glucoside, and cyanidin 3,5-O-diglucoside, which may account for the color reaction in the roots and indirectly cause intrinsic response to stress of Yugu2, were increased 3.4-, 5.2- and 7.2- folds, respectively. While the metabolites related to biomass accumulation, such as seven carbohydrates, fifteen amino acids and their derivatives, three vitamins and ten nucleotides and their derivatives, were reduced (Fig. 7D and Table S13).

### Integrative analysis of transcriptomics and metabolomics

**Figure 8:**
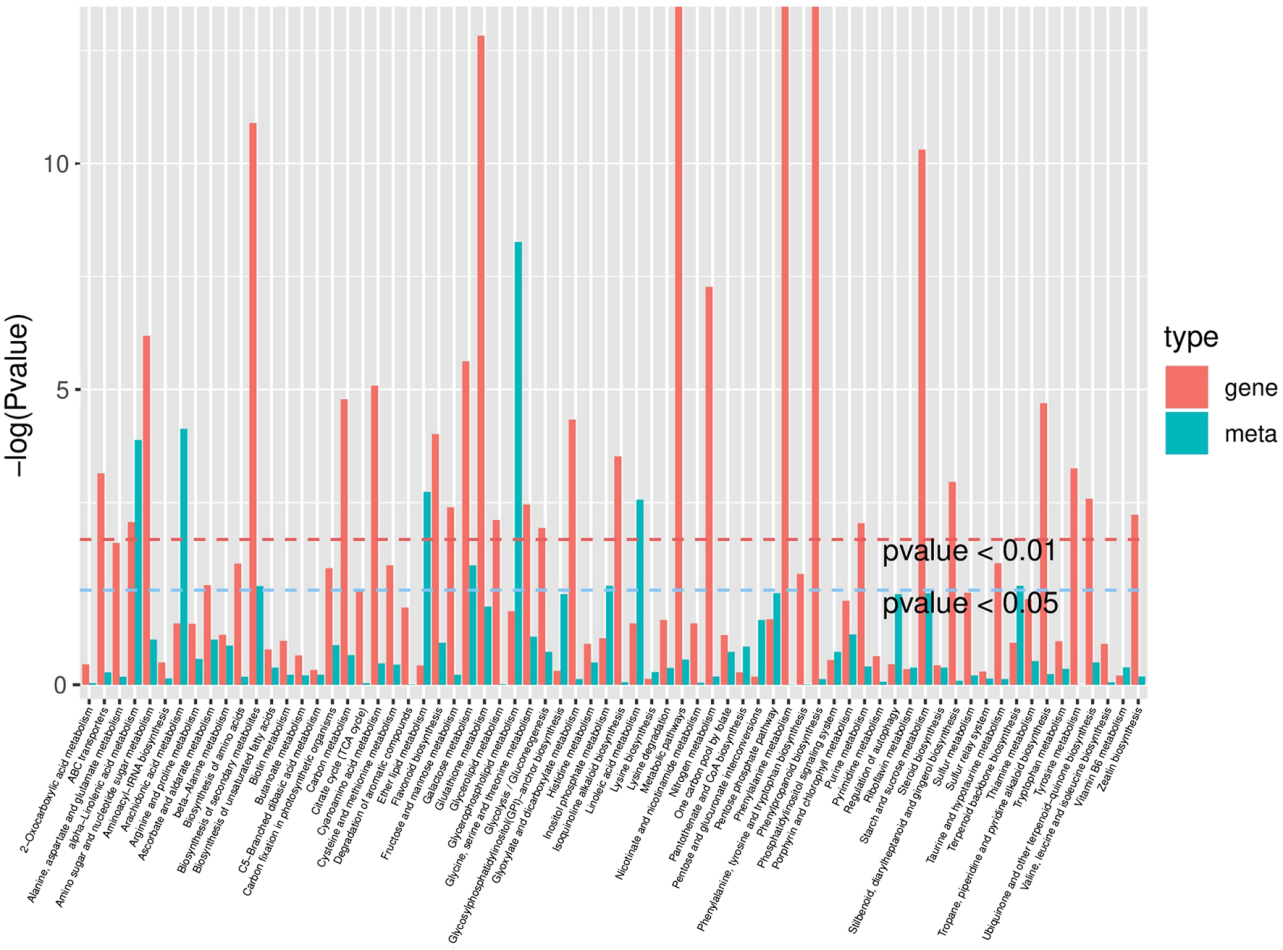
The *p* value histogram of KEGG enrichment analysis for integrated metabolomics and transcriptomics. The x-axis indicates the enriched metabolic pathways, the y-axis represents -log(*P *value), red represents the enriched *p* value of DEGs, and green represents the enriched *p* value of the differential metabolites.

In order to examine the association between metabolites and genes involved in the same biological process, the comprehensive analysis of metabolomics and transcriptomics was performed, and 70 highly-enriched pathways were derived (Fig. 8 and Table S14). The enriched pathways, such as flavonoid biosynthesis, starch and sucrose metabolism, glutathione metabolism, ascorbate and aldarate metabolism, glycerophospholipid metabolism, and biosynthesis of secondary metabolites, all play important roles in the plant stress response process.

The AsA-GSH cycle is a key pathway in plant cell protection against oxidative stress^20^. The genes and metabolites participated in this cycle, such as *APX*, *MDAR*, *GST*, oxidized and reduced GSH and AsA, were identified in Yugu2. The one *GPX* and thirty-two *GST* genes were found to be up-regulated (Table S11). The contents of AsA and GSH showed no significant change in root of Yugu2 after germinated under salt stress for 7 d (Table S12). However, the 7 d-old seedlings of Yugu2 had higher level of AsA and GSH in root than that of An04 after been treated with salt stress for 0 h, 24 h and 48 h (Fig. 5). The increase of lipid biosynthesis gene such as *GPAT* leads to the accumulation of lysophospholipids, which could affect as minor membrane components and signaling mediators under salinity. Seven genes encoding GPAT and twenty-six lysophospholipids were induced in Yugu2 upon salt stress (Table S11).

**Figure 9:**
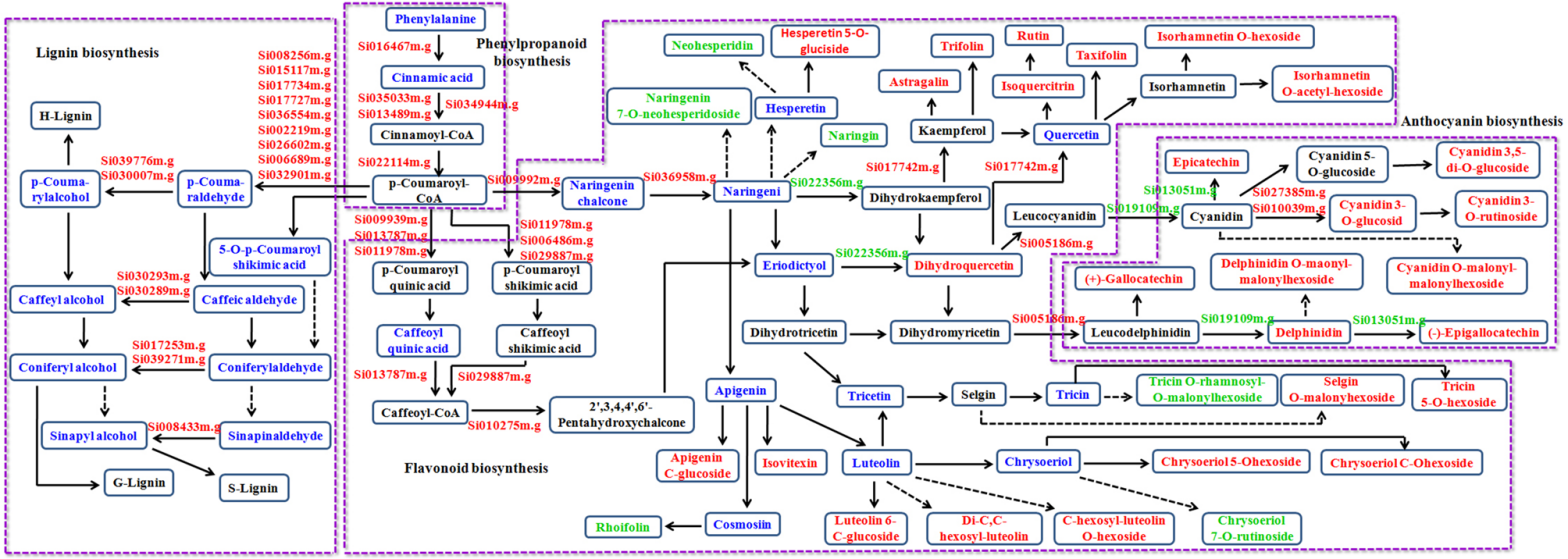
Schematic diagram of DEGs and metabolites in phenylpropanoid, flavonoid, and lignin biosynthetic pathways in Yugu2 root under salinity. The red color represents the up-regulated DEGs and metabolites, whereas the green color represents the down-regulated DEGs and metabolites. The blue color represents the identified metabolites in metabolomics with no significantly changes in contents. The dotted arrows indicate that there are multi-steps between the two metabolites. Microsoft Power Point 2007 was used to create this Figure. *Source*: Kanehisa et al. ^53^.

The phenylpropanoid, flavonoid and lignin biosynthesis pathways were significantly enriched, and total of 33 genes and 27 metabolites were increased (Fig. 9 and Table S15). The genes encoding key enzymes such as shikimate O-hydroxycinnamoyl transferase (HST, Si013787m.g), chalcone-flavonone isomerase (CHI, Si036958m.g), and chalcone synthase (CHS, Si009992m.g) showed 11.8-, 8.9- and 4.2- folds up-regulated, respectively. Simultaneously, large-scale up-regulation of key enzymes encoding genes of the phenylpropanoid, flavonoids biosynthetic pathways could well explain the over-accumulation of flavonoids and anthocyanins (Fig. 9 and Table S15), which in turn enhanced the response abilities of foxtail millet that cope with the oxidative stress of salinity. The biosynthesis of monolignols also commences with phenylalanine and proceeds through the phenylpropanoid pathway. Lignin biosynthesis-related accumulation could maintain cell wall integrity and prevent water loss. Nine genes encoding cinnamoyl-CoA reductase (CCR) and seven genes encoding cinnamyl alcohol dehydrogenase (CAD) were up-regulated in Yugu2 under salt stress. The contents of nine metabolites in this pathway were identified, and the contents of p-coumaroylshikimic acid, caffeyl alcohol and sinapyl alcohol were also found increased by 1.8-, 1.6- and 1.6 folds, respectively (Fig. 9 and Table S15).

## Discussion

It is of great significance to elucidate the regulatory network of salt response in plants due to the threat of salinity imposed to the crops production worldwide. The availability of various germplasms, with the fast-developing techniques, have afforded admirable opportunities to unveil the molecular and metabolic basis of genomic and phenotypic variability of plants under salinity. Plants are heavily depended on roots for adaptive responses to environments and soil reserves, especially important when soil resources are either depleted or inaccessible due to a multitude of stresses^2^.

As reported^21^, *Thellungiella* is the halophyte model for functional genomics. Curiously, very few genes are induced by salt in this plant, while many genes are constitutively expressed at a level higher than that of *Arabidopsis* under salinity, which may be key factors for salt resistance^21^. Similarly, fewer DEGs were identified in salt-tolerance cultivar Yugu2 compared with salt-sensitive cultivar An04 under salinity (Fig. 2A). Pre-stress, the expression of genes in certain pathways, such as phenylpropanoid biosynthesis, phenylalanine metabolism, glutathione metabolism, and biosynthesis of secondary metabolites were significantly higher in Yugu2 (Fig. S5 and Table S8). It is consistent with Yugu2 possessing higher antioxidant enzymes activities and non-enzymatic antioxidant contents (Fig. 5B–E). To sum up, the constitutively expressed genes are dominant in variety characters formation.

### Ion homeostasis is critical for protection of plants from ion toxicity

Salt stress primarily leads to ion toxicity. The SOS pathway is vital for the regulation of plant Na^+^/K^+^ homeostasis via SOS1, which is a key element for Na^+^ efflux^6^. Plant type I H^+^-PPases (AVP1) energized vacuolar Na^+^/H^+^ antiporters are crucial for vacuolar sodium sequestration. Over-expression of *AtAVP1* genes in *Arabidopsis*, barley (*Hordeum vulgare* L.) and other crops presented increased salinity tolerance and higher cereal crop productivity in saline fields^22^. And further, many plants suffer Na^+^ toxicity mainly due to the over-accumulation of Na^+^ in the shoot. To cope with this, OsHKT1;5 and AtHKT1;1 could mediate Na^+^ exclusion from leaves via Na^+^ removal from the xylem sap^23^. Over-expression of HKT1;1 specifically in root stele could reduce root-to-shoot transfer of Na^+^ and shoot Na^+^ accumulation, and enhance the whole-plant salinity tolerance^24^. Here the ortholog of *SOS2* (Si035799m.g and Si029714m.g), *SOS3* (Si026897m.g and Si011148m.g), *AVP1* (Si000395m.g), and *HKT* (Si001234m.g) all showed higher expression levels in Yugu2. Reversely, the K^+^ channel encoding genes, such as *AKT1* (Si000254m.g), *HAK* (Si012185m.g and Si034300m.g), and *KOR* (Si005846m.g), expressed extremely higher in An04. K^+^ functions as enzyme activator to promote metabolism and accelerates the translocation of photosynthates for plant growth or storage in roots or fruits^25^. Considering the K^+^ channel is in charge of pump K^+^ into cell, and the higher K^+^ content detected in An04 cultivar (Fig. 1F), it is reasonable explanations for the greater biomasses and higher grain yields of An04 under well-watered condition as previous reported^9^.

### Ca^2+^ signal and kinase cascades under salinity

Recent report has shown that calmodulin-binding transcription activator 6 (CAMTA6) is a key negative regulator of most salt-responsive genes and ion homeostasis during early germination^26^. In our results, one *CAMTA* (Si000282m.g) gene was identified, and its decrease also confirmed the previous conclusion that Yugu2 is less sensitive to salt.

The Ca^2+^ signal could induce the increase of the ABA concentration in plant cell, and trigger initiation of the transcriptional responses^4^. The higher levels of *CNGC* and *NCED1* in YG2TvsAn04T (Table S11) maybe also indicated that there are more rapid and intense responses in Yugu2 under salinity. Previous reports showed AtMEKK1-MKK2-MPK4/MPK6 cascade is critical in stress signaling responses in *Arabidopsis*^27^. MKK9-MPK3/MPK6 cascade could regulate salt sensitivity via controlling ethylene and camalexin biosynthesis^28^. MPK6 also participate in phosphatidic acid (PA) mediated salt-stress signaling by activating SOS1^7^. The salinity induced Ca^2+^ in cytosol, which in turn leads to produce superoxide in apoplast, and the death signal of programmed cell death (PCD) that transmitted by ROS also depends on MAPK signal pathway. Here several MAPK cascade related DEGs (including MAPKKKs, MAPKKs, and MAPKs) have also been identified, and similar expression patterns in both varieties indicated important roles in salt signaling and response in foxtail millet. Considering the SOS signal pathway and MAPK cascade have not ever been reported in foxtail millet, identified and verified DEGs involved in these pathways here suggest that they are conservative and constitutive in salt resistance response in foxtail millet.

### ROS signal and scavenging

Salinity also cause oxidative stress and ROS-associated injury in plants. To deal with this problem, plants evolved two main strategies to eliminate excessive ROS, the enzymatic degradation and deoxidization by small molecules such as AsA, GSH, flavonoids, carotenoids and phenolic compounds^29^. Over-expression of GST/GPX from different species via genetic manipulation was observed to enhance tolerance toward abiotic stress through assisting peroxide scavenging and oxidative stress metabolism^30^. Here the higher expression levels of *GST*, *GPX* and *POD* in Yugu2 (Table S11), in addition with higher contents of non-enzymatic antioxidant, such as AsA, GSH and flavonoids (Figs. 5 and 9), all conferred Yugu2 possessing stronger antioxidant capacity.

### Transcription factors (TFs) participat in salt response

Partial of TFs have been reported to participate in salt response. As mentioned above, phenolic compounds such as flavonoids produced as the main anti-salinity effectors. The transcription of flavonoids synthesis genes are regulated by a series of TFs. R2R3MYB combined with bHLH and WD40 proteins could form MBW (MYB-bHLH-WD40) transcriptional activation complex which further regulate the expressions of anthocyanin biosynthetic genes in most plants^31^. Genes encoding *CHS*, *CHI*, flavanone 3-hydroxylase (F3H), and dihydroflavonol-4-reductase (DFR) that are related to different flavonoid biosynthetic steps are usually regulated by MYB and bHLH families. The R2R3 MYB transcription factors, such as PAP1 (MYB75), PAP2 (MYB90), TT2 (MYB123), play a central role in activating genes of the anthocyanin biosynthesis pathway^31^. In our study, five R2R3 MYB TFs increased in YG2TvsAn04T and YG2TvsYG2C (Fig. S6). The identified DEGs of TFs might be regulators for activating the transcription of the anthocyanin biosynthetic genes, and further contribute to flavonoids elevating and salt resistance improving of foxtail millet.

### The role of hormones in response to salt

Previous reports showed that salt stress represses root meristem growth through inhibiting PINFORMED (PIN) expression and stabilizing IAA17, thereby decreasing auxin levels and suppressing auxin signaling, respectively^12^. Suppression of root elongation by glucose and copper were also mediated by modulation of PIN1 expression^32,33^. In our research, five PIN genes were also evidently repressed in both An04 and Yugu2, which may result in reduction of auxin accumulation and growth inhibition in root. However, this phenotype of root inhibition was remission in Yugu2 as the higher expression levels of six *IAA* genes identified in YG2TvsAn04T (Fig. 1A,D, Table S11). Simultaneously, exogenous auxin could partially rescue the root inhibition under salinity (Fig. S7). Stress-induced DELLA accumulation could restrain growth and enhance the tolerance to adversity by mean of up-regulation of ROS detoxification enzymes under stress^34^. The decreased GA synthesis related genes and the increased deactivation related genes might ultimately reduce the bioactive GA levels in foxtail millet, which in turn resulted in the accumulation of DELLA proteins and then restrain root growth. The higher expression level of DELLA genes (Si009972m.g and Si000959m.g) in YG2TvsAn04T, in addition with higher expression level of ROS scavenging related genes (Table S11), which conferred Yugu2 possessing higher salt tolerance than An04. However, the dual function of DELLA proteins requires further attention.

### Metabolites accumulation and function under salinity

As the final recipient of biological information flow, the metabolome investigation are the main readouts of gene vs environment interactions and represent the sum of all the levels of regulation in between genes and enzymes under stress^35^.

A mountain of metabolites such as compatible solutes, nitrogen-containing compounds, antioxidants and signal macromolecules were also found to be regulated in salt-tolerance variety^36^. Over twenty-eight class of metabolites were detected in Yugu2 upon salinity stress, especially the contents of flavonoids (Table S13 and Fig. 7D). Flavonoids are one of the most bioactive plant secondary metabolites, and function as key antioxidants in stressed plant^29^. A wide range of abiotic stresses, such as salinity, drought, and UV light, all could induce the expression of many flavonoid biosynthetic genes and enhance the accumulation of flavonoids in diverse species^37,38^. Recent report has shown that phosphorylated GmMYB173 regulates dihydroxy B-ring flavonoids syntheses via transcriptional control of *GmCHS5*, and thus contributes to soybean salt tolerance^39^. Integrated analysis of metabolomic and transcriptomic data of foxtail millet roots in this research showed that 17 flavonoid biosynthesis related genes were significantly up-regulated 2- to 11-fold in Yugu2 under salinity (Table S16). In keeping with genes expressions, the over-accumulation of 27 flavonoids metabolites were obtained (Fig. 9 and Table S15). In brief, the elevated expression levels of antioxidant enzyme genes and the enhanced flavonoids accumulation in salt tolerant Yugu2 indicated that flavonoids are important to the formation of resistance characters of foxtail millet.

The innovative finding in this research is that Lysophospholipids were increased greatly in seedling roots of Yugu2 under salinity. Lysophospholipids are bioactive phospholipid and found as minor membrane components and signaling mediators. Abiotic and biotic stress, such as wounding, freezing, elicitors and pathogen infection, could induce the accumulation of lysophospholipids^40,41^. Recent report provides evidence that H^+^-ATPases may act as potential receptors for lysophospholipids recognition, and then initiate signaling pathways in plants^40,42^. Lysophospholipids function as signal transducer participating in regulation of auxin responses and activation of tonoplast H^+^/Na^+^-antiporter to generate a cytoplasmic pH shift^43^. The over-accumulation of lysophospholipids in foxtail millet under salinity here also indicated that it participates in salt stress signal transduction as a signal transduction component. As the function of this metabolite in stress response is rarely reported in plants, it deserves further investigation to uncover its specific role in foxtail millet under salt stress.

The biomass related metabolites, such as carbohydrates, amino acid and nucleotide, were found to be down-regulated in Yugu2 under salinity which appeared different from previous reports. The decrease of these metabolites may due to their further use for carbon skeletons providing and precursors of protective secondary metabolites synthesizing.

Considering the limitations of sample preparation and lack of specific stands for metabolites, such as cuticular wax, cellulose, etc., the shortcomings and deficiencies were still existed in integrate analysis. Quantitative proteomics and phosphoproteomics analysis were also undergoing in trying to decipher the deeper and transient salt response mechanisms of foxtail millet.

## Conclusion

**Figure 10:**
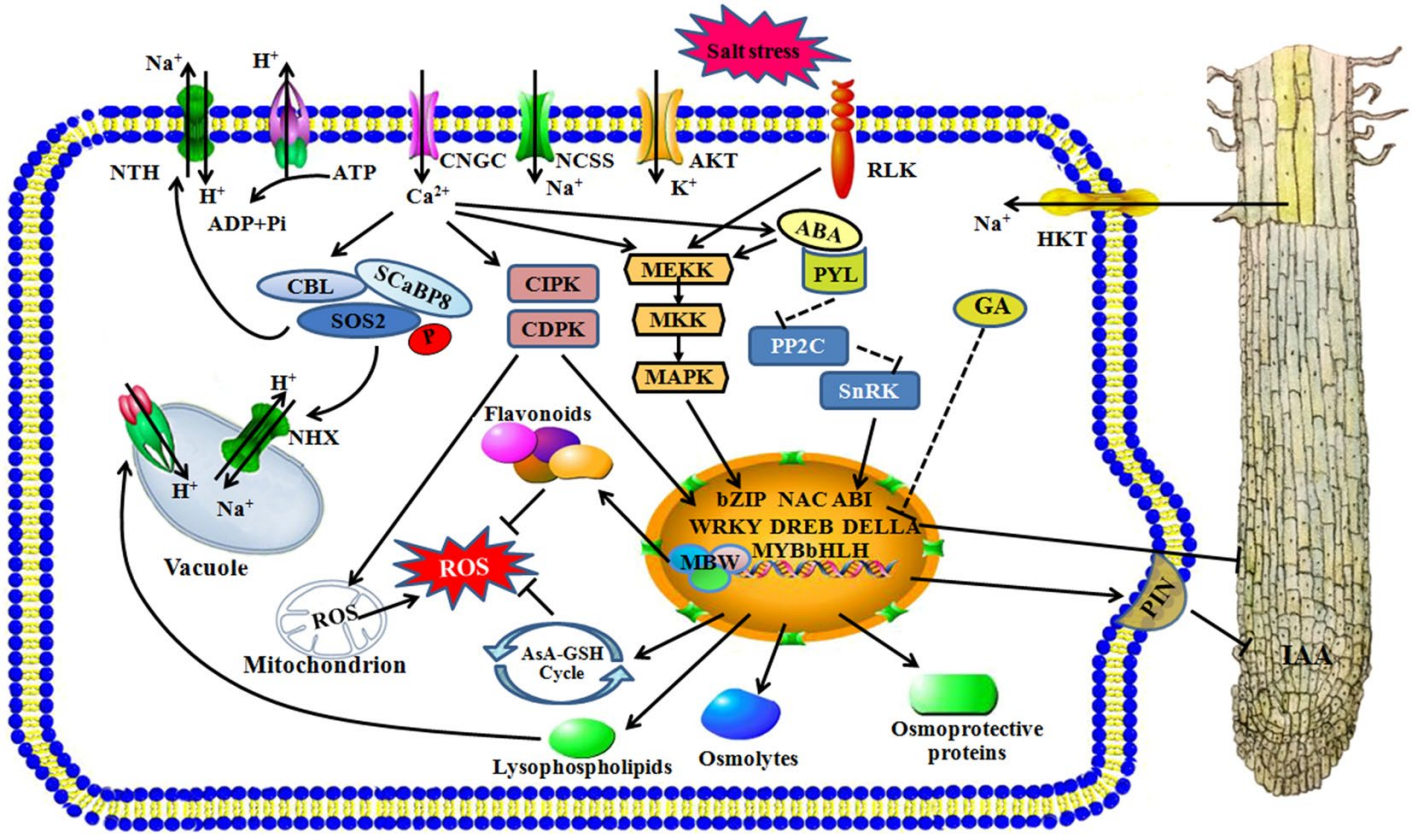
The schematic presentation of salt-responsive mechanisms in root of foxtail millet. Abbreviations: NSCC: nonselective cation channel, CNGC: cyclic nucleotide-gated ion channel, RLK: receptor-like protein kinase, HKT: high-affinity K^+^ transporter, NHX: Na^+^/H^+^ exchanger, CDPK: calcium-dependent protein kinase, CIPK: CBL-interacting protein kinase, SOS: salt overly sensitive, SCaBP8: SOS3-Like calcium binding protein 8, NHX: Na^+^/H^+^ exchanger, MAPK: mitogen-activated protein kinase, MKK: mitogen-activated protein kinase kinase, MEKK: mitogen-activated protein kinase kinase kinase, PP2C: protein phosphatase 2C, DREB: dehydration-responsive element-binding proteins, ERF: ethylene-responsive factor, GA: gibberellin, ABA: abscisic acid, PIN: PINFORMED, MBW: MYB-bHLH-WD40 transcriptional activation complex. Microsoft Power Point 2007 was used to create this Figure.

Here several classical mechanisms like SOS pathways and MAPK cascade are proved to be conservative existed in foxtail millet, and some generally accepted metabolites like flavonoids are essential in participating in coping with stress. The regulation model of foxtail millet under salinity could be summarized as follows. Salt stress results in increased Na^+^ content, which further provokes cytosolic Ca^2+^ signal. The ion channels and signaling pathways were activated and ultimately resulted in activation of cellular detoxification mechanisms. The phytohormones such as IAA and GA which dominated in root elongation were also infected under salinity (Fig. 10). It is noteworthy that partial metabolites such as lysophospholipids were identified in response to salinity, which represent that there may exist diverse or new mechanisms in foxtail millet to deal with salinity. Considering as a whole, the DEGs and metabolites appeared in this research need further verification, and the candidate factors could be defined as associated with stress tolerance and could serve as accurate markers for salt-tolerant crop selection in breeding programs.

## Metarials and methods

### Plant materials, growth conditions and physiological measurements

Seeds of two foxtail millet cultivars An04 and Yugu2 were surface sterilized. And then seeds were germinated in petri dish lined with filter papers moistened either with distilled water alone for control treatment or with 150 mM NaCl solutions for salt stress treatment. The seeds were germinated in chamber under 30/25 °C day/night cycle with a 14-h photoperiod for seven days. The roots were sampled from the control and salt-treated seedlings with three independent biological replications for each sample. The roots were washed gently with sterile water and then frozen in liquid nitrogen for RNA-Seq and metabolomics analysis.

For physiological measurements, the seeds were surface sterilized and hydroponically cultivated. The 7 day old seedlings were then treated with 150 mM NaCl solutions, and the roots were sampled after been treated for 24 h and 48 h respectively. The samples were used for physiological measurements with three biological replicates. The physiological parameters, such as histochemical detection of H_2_O_2_ and O_2_^-^, antioxidant enzyme activity, and the GSH and AsA contents, were measured as described previously^44^. The contents of K^+^ and Na^+^ were determined following the methods of Liu et al. ^45^. The surface sterilized seeds of two foxtail millet cultivars An04 and Yugu2 were germinated under salt stress plus 20 nM IAA treatment for 7 days, and the length of roots were measured and calculated.

### RNA library construction and sequencing

Total RNA was isolated from the seedling roots of foxtail millet using Total RNA Isolation Kit (China) according to the manufacturer’s protocol. RNA integrity was checked by the RNA Nano 6000 Assay Kit of Bioanalyzer 2100 system (Agilent Technologies, CA, USA). Sequencing libraries were generated according to Xiong et al. (2017)^46^.

The RNA-seq libraries were sequenced on an Illumina Hiseq platform X ten as previous reports^9,45,46^ and 125 bp/150 bp paired-end reads were generated. Raw data (raw reads) of fast q format were firstly processed through in-house perl scripts. All the downstream analyses were based on the clean data with high quality.

Index of the reference genome was built using Hisat2 v2.0.5, and paired-end clean reads were aligned to the reference genome using Hisat v2.0.5. Feature Counts v1.5.0-p3 was used to count the reads numbers mapped to each gene. And then FPKM of each gene was calculated based on the length of the gene and the read count mapped to this gene. Differential expression analysis of each comparison was performed using the DESeq2 R package (1.16.1). Genes had a fold change value ≥ 2 (|Log2 fold change|≥ 1) between two groups and with an adjusted *P* value < 0.05 found by DESeq were considered to be differentially expressed. Functional enrichment analyses of DEGs were performed as previous reports^46,47^.

### Validation of DEGs using qRT-PCR

The expressions of twenty randomly selected DEGs were examined by qRT-PCR^11^. And the relative gene expression levels were analyzed as described^48^. The primers are listed in Supplementary Table S16.

### Metabolomics analysis of seedling roots of foxtail millet based on LC–MS data

The sample preparation and metabolomics analysis were performed as previously described^49–51^. The sample extracts were analyzed by an LC–ESI–MS/MS system (HPLC, Shim-pack UFLC SHIMADZU CBM30A system, and MS, Applied Biosystems 6500 Q TRAP). The analytical conditions were performed according to Wang et al. (2018)^50,51^. LIT (linear ion trap) and triple quadrupole (QQQ) scans were captured via triple quadrupole-linear ion trap mass spectrometer (Q TRAP), API 6500 Q TRAP LC/MS/MS System. The mass spectrometry conditions were performed according to Wang et al. (2018)^50,51^.

### Metabolites identification and quantification

Metabolites identification were carried out based on the MWDB V2.0 Database of Wuhan Metware Biotechnology Co., Ltd. (Wuhan, China), and the metabolites information public database, such as KNAPSAcK (https://kanaya.naist.jp/KNApSAcK/), METLIN (https://metlin.scripps.edu/index.php), HMDB (https://www.hmdb.ca/), MoToDB (https://www.ab.wur.nl/moto/), and MassBank (https://www.massbank.jp/). The metabolites were quantified using multiple reaction monitoring (MRM) of triple quadrupole mass spectrometry according to Chen et al.^51^. Peak area integration was performed on the obtained metabolite mass spectral peaks, and the mass spectral peaks of the metabolites in different samples were integrated. Hierarchical clustering analysis (HCA) and principle component analysis (PCA) and partial least squares discriminant analysis (PLS–DA) were carried out^50,51^. The threshold of variables determined to be important in the projection (VIP) scores ≥ 1.0 together with fold change ≥ 2 or ≤ 0.5 was adopted to assess significant different metabolites. The Kyoto Encyclopedia of Genes and Genomes (KEGG) database was used to link differential metabolites and DEGs to metabolic pathways^52^.

### Statistical analysis

Statistical analysis was carried out via SigmaPlot Version 11.0, SPSS13.0, and Excel 2007 software.

## Supplementary information

Supplementary Table S1.

Supplementary Table S2.

Supplementary Table S3.

Supplementary Table S4.

Supplementary Table S5.

Supplementary Table S6.

Supplementary Table S7.

Supplementary Table S8.

Supplementary Legend.

Supplementary Table S9.

Supplementary Table S10.

Supplementary Table S11.

Supplementary Table S12.

Supplementary Table S13.

Supplementary Table S14.

Supplementary Table S15.

Supplementary Table S16.

Supplementary Figure S1.

Supplementary Figure S2.

Supplementary Figure S3.

Supplementary Figure S4.

Supplementary Figure S5.

Supplementary Figure S6.

Supplementary Figure S7.

## Data Availability

The raw data generated under this study have been deposited in the NCBI database with the Accessions Number of PRJNA545871.
