# Supplementary material for: Integrative analyses of transcriptomics and metabolomics upon seed germination of foxtail millet in response to salinity
Source: Sci Rep. 2020 Aug 12;10:13660. doi: 10.1038/s41598-020-70520-1 (PMC7423953; doi:10.1038/s41598-020-70520-1)
Supplement: Supplementary file 9 — Supplementary Legend. [file 41598_2020_70520_MOESM9_ESM.docx]

**Transcriptomics and Metabolomics Analysis to Unveil Complex Mechanisms underlying Salinity Response of Foxtail Millet (*Setaria italica* L.) Plantlet**

Jiaowen Pan^1^, Zhen li^1^, Shaojun Dai^6^, Hanfeng Ding^3, 4^, Qingguo Wang^1^, Xiaobo Li^5^, Guohua Ding^5^, Pengfei Wang^7^, Yanan Guan^2, 4*^, Wei Liu^1, 4*^

^1^Biotechnology Research Center, Shandong Academy of Agricultural Sciences; Key Laboratory of Genetic Improvement, Ecology and Physiology of Crops, Jinan 250100, Shandong, PR China

^2^Crop Research Institute, Shandong Academy of Agricultural Sciences, Shandong engineering laboratory for featured crops, Jinan 250100, Shandong, PR China

^3^Shandong Center of Crop Germplasm Resources, Shandong Academy of Agricultural Sciences, Jinan 250100, Shandong, P R China

^4^College of Life Sciences, Shandong Normal University, Jinan 250014, Shandong, PR China

^5^School of Life Science and Technology, Harbin Normal University, Harbin 150025, Heilongjiang, PR China

^6^Development Center of Plant Germplasm Resources, College of Life and Environmental Sciences, Shanghai Normal University, Shanghai 200234, PR China

^7^Shandong Academy of Grape; Shandong Engineering Research Center for Grape Cultivation and Deep-processing, Jinan 250100, Shandong, PR China

***** Corresponding authors:

Wei Liu: E-mail: [wheiliu@163.com](mailto:wheiliu@163.com), Tel: +86-531-66658133

Yanan Guan: E-mail: [yguan65@163.com](mailto:yguan65@163.com); Tel: +86-531-66658115

**Supplementary data:**

**Figure S1.** The relative germination rate, root and seedlings lengths of 14 varieties of foxtail millet under salt stress. Each experiments repeat for three times.

**Figure S2.** Gene expression analyses of Yugu2 and An04 subjected to salt stress. A FPKM distribution of gene expressions for all samples. B Violin plots of FPKM distribution.

**Figure S3.** Gene ontology (GO) analysis of DEGs. The x-axis means the number of DEGs, and the y-axis indicates the GO terms.

**Figure S4.** Scatter plot of top 20 KEGG pathways for DEGs in YG2TvsYG2C and An04TvsAn04C.

**Figure S5.** Scatter plot of top 20 KEGG pathways for DEGs in YG2CvsAn04C .

**Figure S6.** Protein sequences alignment of five DEGs encoding R2R3-MYB TFs. The R2R3 motif is indicated at the top.

**Figure S7.** A The phenotypes of foxtail millet seeds germinated and grown in sterile water, 150 mM NaCl, and 150 mM NaCl plus 20 nM IAA solution for 7 days. B The root length of 7-day seedlings of Yugu2 and An04 grown in sterile water, 150 mM NaCl, and 150 mM NaCl plus 20 nM IAA solution. The SigmaPlot Version Version 11.0 was used to draw histograms in B.

**Table S1.** Expression profiles and gene annotations of DEGs in RNA-seq.

**Table S2.** GO enrichment analysis of DEGs in YG2TvsYG2C.

**Table S3.** GO enrichment analysis of DEGs in An04TvsAn04C.

**Table S4.** GO enrichment analysis of DEGs in YG2CvsAn04C.

**Table S5.** GO enrichment analysis of DEGs in YG2TvsAn04T.

**Table S6.** KEGG enrichment analysis of DEGs in YG2TvsYG2C.

**Table S7.** KEGG enrichment analysis of DEGs in An04TvsAn04C.

**Table S8.** KEGG enrichment analysis of DEGs in YG2CvsAn04C.

**Table S9.** KEGG enrichment analysis of DEGs in YG2TvsAn04T.

**Table S10.** GO enrichment analysis of DEGs in each sub-clusters of K-means.

**Table S11.** Key genes responsible for salt tolerance in the Yugu2.

**Table S12.** 720 metabolites were identified in Yugu2 root.

**Table S13.** 251 differential metabolites in Yugu2 root under salt stress.

**Table S14.** KEGG enrichment analysis of integrated metabolomics and transcriptomics in Yugu2 under salinity.

**Table S15.** Metabolites and DEGs in phenylpropanoid, flavonoid, and lignin biosynthetic pathways in Yugu2 root under salt stress.

**Table S16.** Oligonucleotide primers used in qRT-PCR.
