# Supplementary material for: Integrative analyses of transcriptomics and metabolomics upon seed germination of foxtail millet in response to salinity
Source: Sci Rep. 2020 Aug 12;10:13660. doi: 10.1038/s41598-020-70520-1 (PMC7423953; doi:10.1038/s41598-020-70520-1)
Supplement: Supplementary file 17 — Supplementary Table S16. [file 41598_2020_70520_MOESM17_ESM.docx]

Table S1 List of the sequences of the primers used in qRT-PCR.

| Gene ID | Primer | Sequence (5'-3') | Annealing temperature | Amplicon size |
| --- | --- | --- | --- | --- |
| Si000254m.g | AKT-S1 | GTTGGAGAAGGATAGACA | 57℃ | 110bp |
|  | AKT-A1 | CAGCAAGAAGATAGTAGAAG |  |  |
| Si022119m.g | AMY-S1 | TGATTTGTTTCTCTGCTA | 57℃ | 107bp |
|  | AMY-A1 | CATTCTGTGTATAATTCGTA |  |  |
| Si013722m.g | CIPK-S1 | GCTAGTCGTAGGGTACAGTTT | 57℃ | 110bp |
|  | CIPK-A1 | CCATGAGGGTCGAATGCT |  |  |
| Si011148m.g | CML-S1 | CGTGCCGTTGATCCATAT | 57℃ | 81bp |
|  | CML-A1 | GACACAGGTATTGACATGG |  |  |
| Si009413m.g | CSLH-S1 | CCAACCAACAAGGATGAG | 57℃ | 82bp |
|  | CSLH-A1 | CGATTCGATCAGTTCTCTT |  |  |
| Si000959m.g | DELLA-S1 | TCGCTTGTATTGCTCTAT | 57℃ | 149bp |
|  | DELLA-A1 | TCCTCGTTAGTTTAGTTAGT |  |  |
| Si030998m.g | DREB-S1 | CCAACTCAGTCAGAACGA | 57℃ | 90bp |
|  | DREB-A1 | CGCTCATCTCCTTCTTGA |  |  |
| Si008092m.g | G2OX-S1 | CCTATCACATGGCATACG | 57℃ | 80bp |
|  | G2OX-A1 | TCGCTTGTTAGTCACAGT |  |  |
| Si001573m.g | GAOX-S1 | CTCCCCTGCTACAAATAC | 57℃ | 123bp |
|  | GAOX-A1 | TTGGGACACCATGAGATG |  |  |
| Si001160m.g | GPAT-S1 | ATGCGGGTCTACATCAAC | 57℃ | 84bp |
|  | GPAT-A1 | CTTGACGATGAGCCTGAT |  |  |
| Si007180m.g | GST-S1 | CTCTACCTCCACTCCATC | 57℃ | 93bp |
|  | GST-A1 | CTGACTCTCCATACAACAA |  |  |
| Si007373m.g | IAA-S1 | TGTCTCATAATATGGCTTGGCTA | 57℃ | 80bp |
|  | IAA-A1 | ATACACGCACTGGTCTGAT |  |  |
| Si035360m.g | KCS-S1 | ACATCCTCGTCGTCAACT | 57℃ | 80bp |
|  | KCS-A1 | GCGGAGCTTGTACTTGTT |  |  |
| Si000788m.g | MPK8-S1 | ATCTCCACCAAGTCATCA | 57℃ | 157bp |
|  | MPK8-A1 | TTACAGTTAGCATTAGCCAATA |  |  |
| Si003841m.g | MYB-S1 | GATAGTTTCTGGCGTTTC | 57℃ | 91bp |
|  | MYB-A1 | CAGTATGCTCCTAAGGTA |  |  |
| Si024703m.g | NCED-S1 | GACCATGATCCACGACTTC | 57℃ | 81bp |
|  | NCED-A1 | ATCTCGCCGAGCTTGAAC |  |  |
| Si006110m.g | PIN-S1 | ACTTCATCTCCACCAACAA | 57℃ | 107bp |
|  | PIN-A1 | GAGGACGATGAGCTTCTG |  |  |
| Si011925m.g | POD-S1 | CTCAGGTTCAGGTGACAA | 57℃ | 80bp |
|  | POD-A1 | GATTCTTGTAGTAGTTGTTCTCA |  |  |
| Si024914m.g | PP2C-S1 | TGAACTACTGATGGATTCGT | 57℃ | 112bp |
|  | PP2C-A1 | ATCATGCTGTAGCGGAAG |  |  |
| Si018378m.g | PYL-S1 | ATGCCTATCAAGTGGATC | 57℃ | 96bp |
|  | PYL-A1 | ATTAAGCGAAGGTGAAGT |  |  |
| XM_004978702 | SiActin1-S1 | CGCATATGTGGCTCTTGACT | 57℃ | 126bp |
|  | SiActin1-A1 | GGGCACCTAAATCTCTCTGC |  |  |
