# Supplementary figures and images for: Integrative analyses of transcriptomics and metabolomics upon seed germination of foxtail millet in response to salinity
Source: Sci Rep. 2020 Aug 12;10:13660. doi: 10.1038/s41598-020-70520-1 (PMC7423953; doi:10.1038/s41598-020-70520-1)

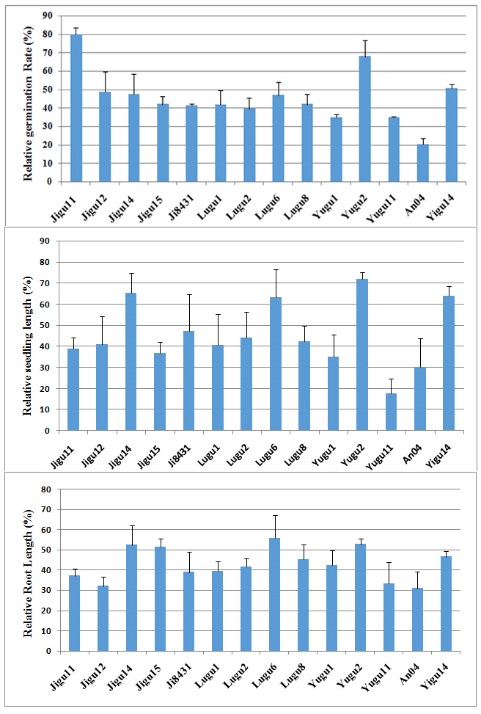

Supplement: Supplementary file 18 — Supplementary Figure S1. [file 41598_2020_70520_MOESM18_ESM.docx]

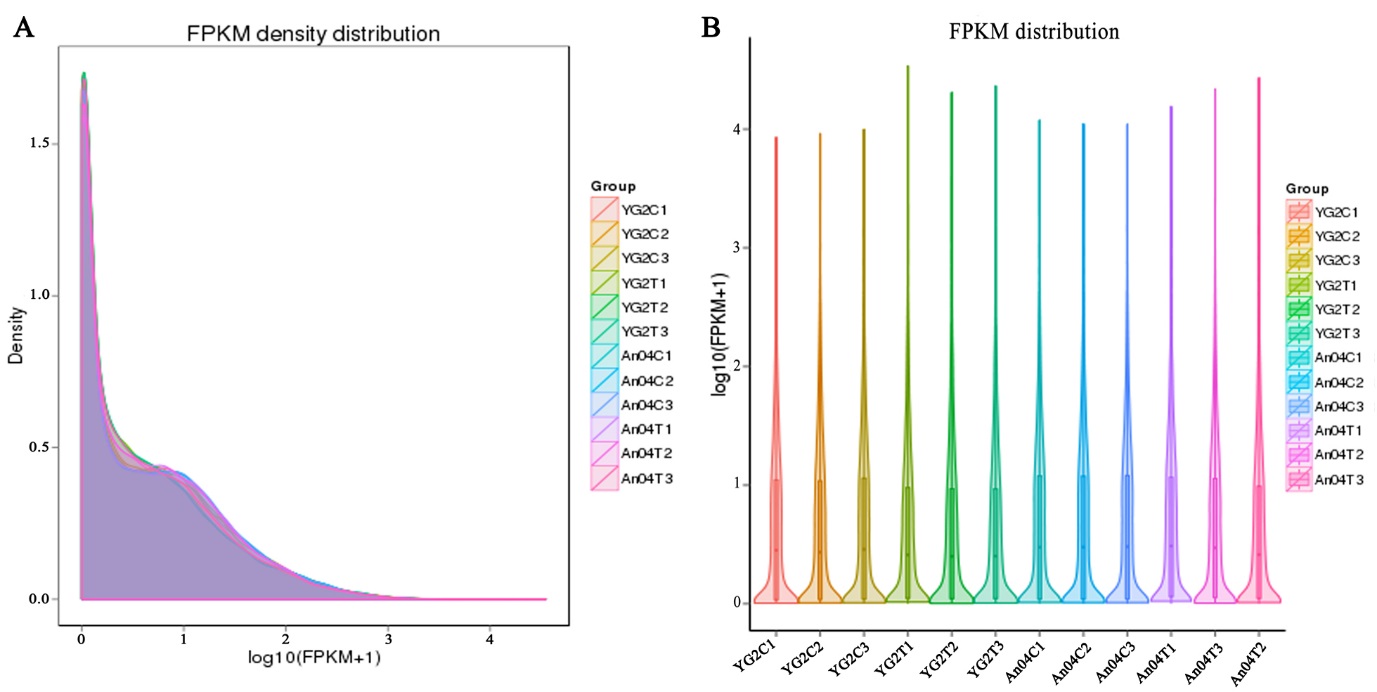

Supplement: Supplementary file 19 — Supplementary Figure S2. [file 41598_2020_70520_MOESM19_ESM.docx]

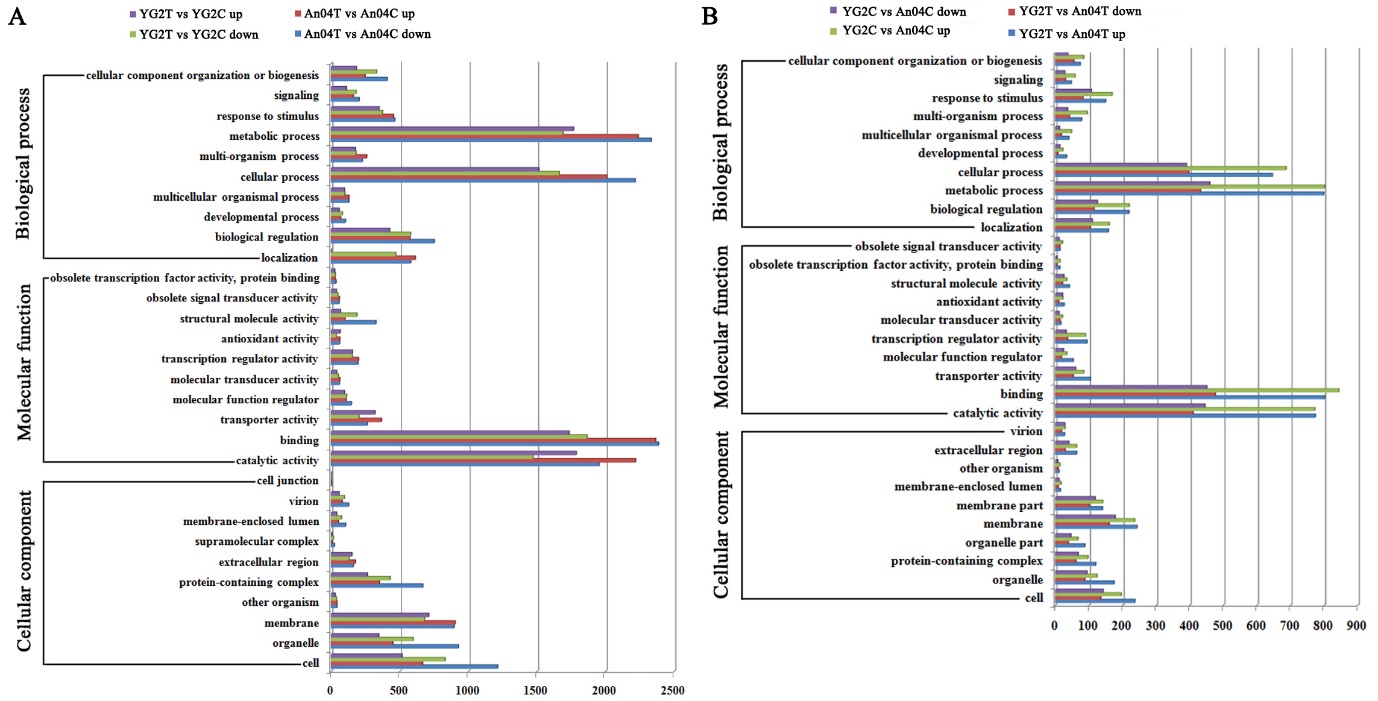

Supplement: Supplementary file 20 — Supplementary Figure S3. [file 41598_2020_70520_MOESM20_ESM.docx]

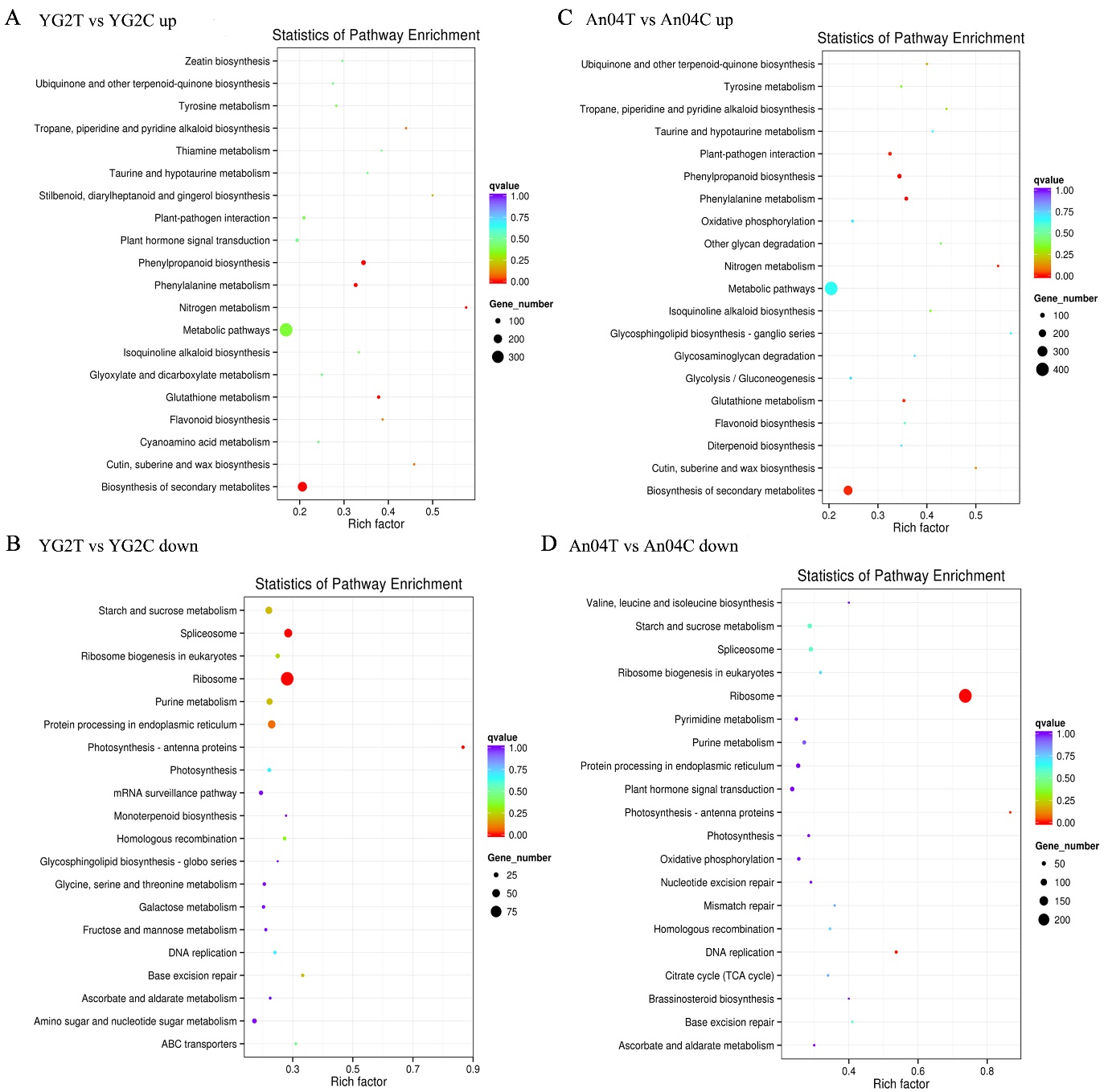

Supplement: Supplementary file 21 — Supplementary Figure S4. [file 41598_2020_70520_MOESM21_ESM.docx]

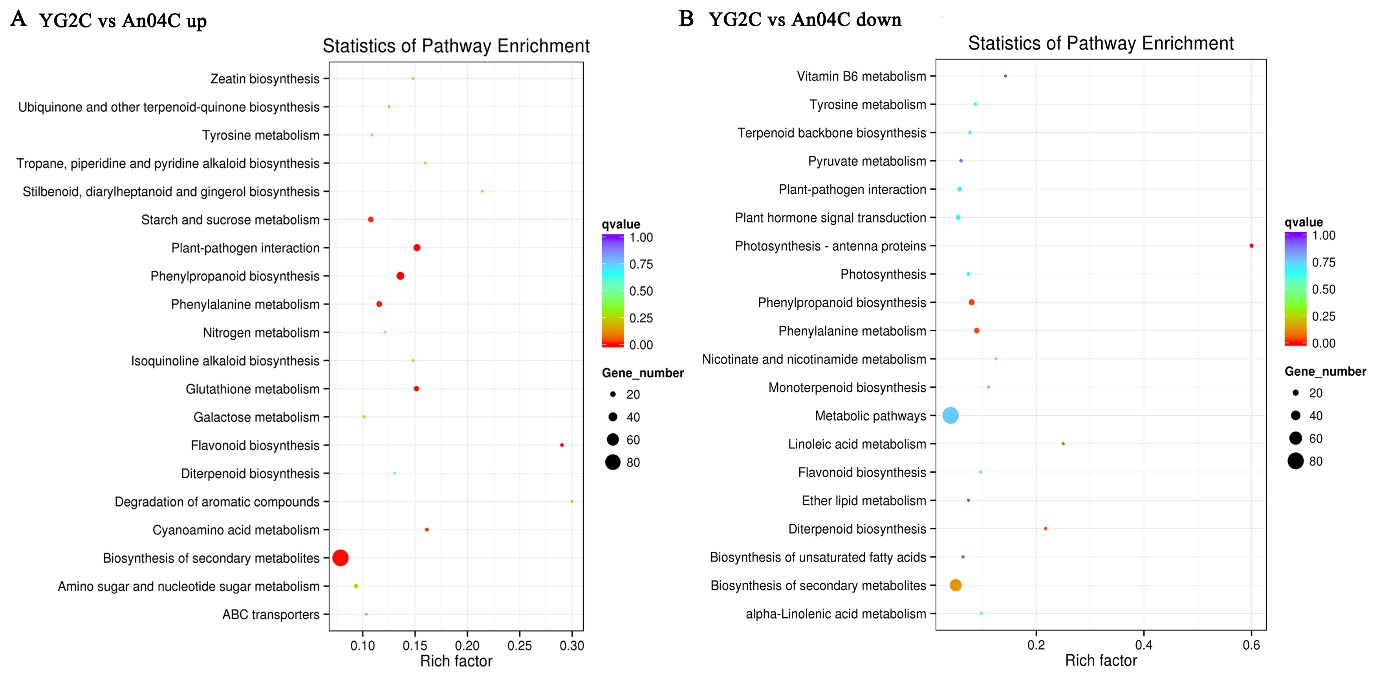

Supplement: Supplementary file 22 — Supplementary Figure S5. [file 41598_2020_70520_MOESM22_ESM.docx]

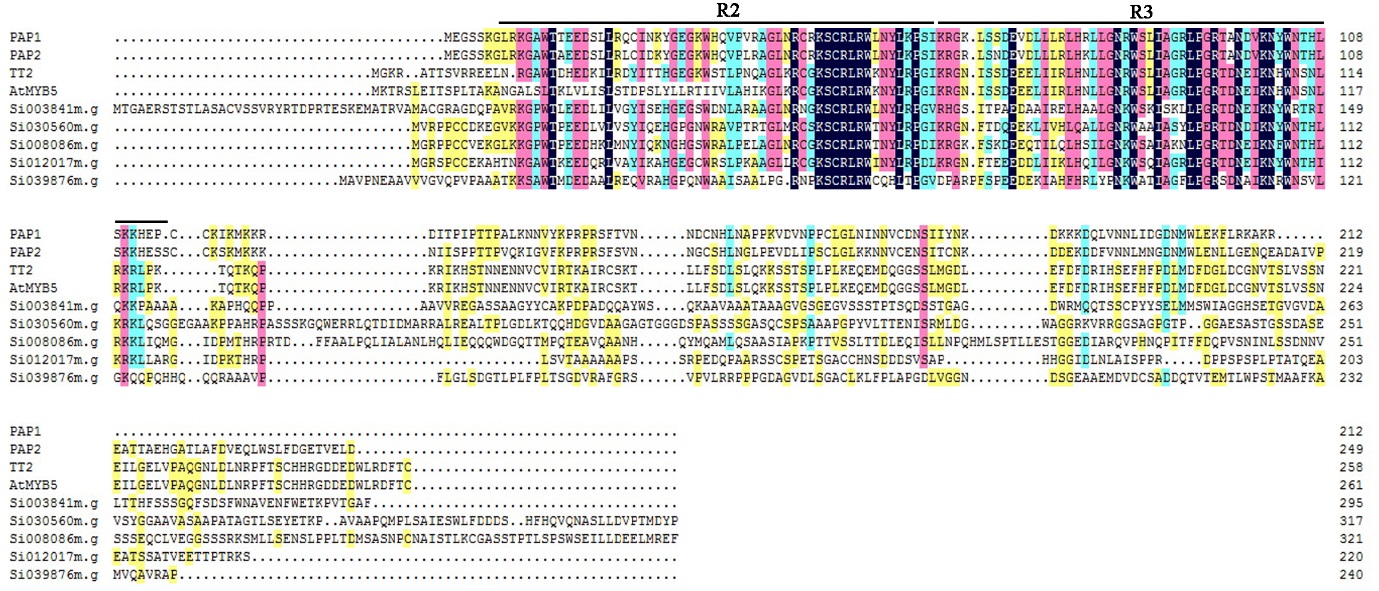

Supplement: Supplementary file 23 — Supplementary Figure S6. [file 41598_2020_70520_MOESM23_ESM.docx]

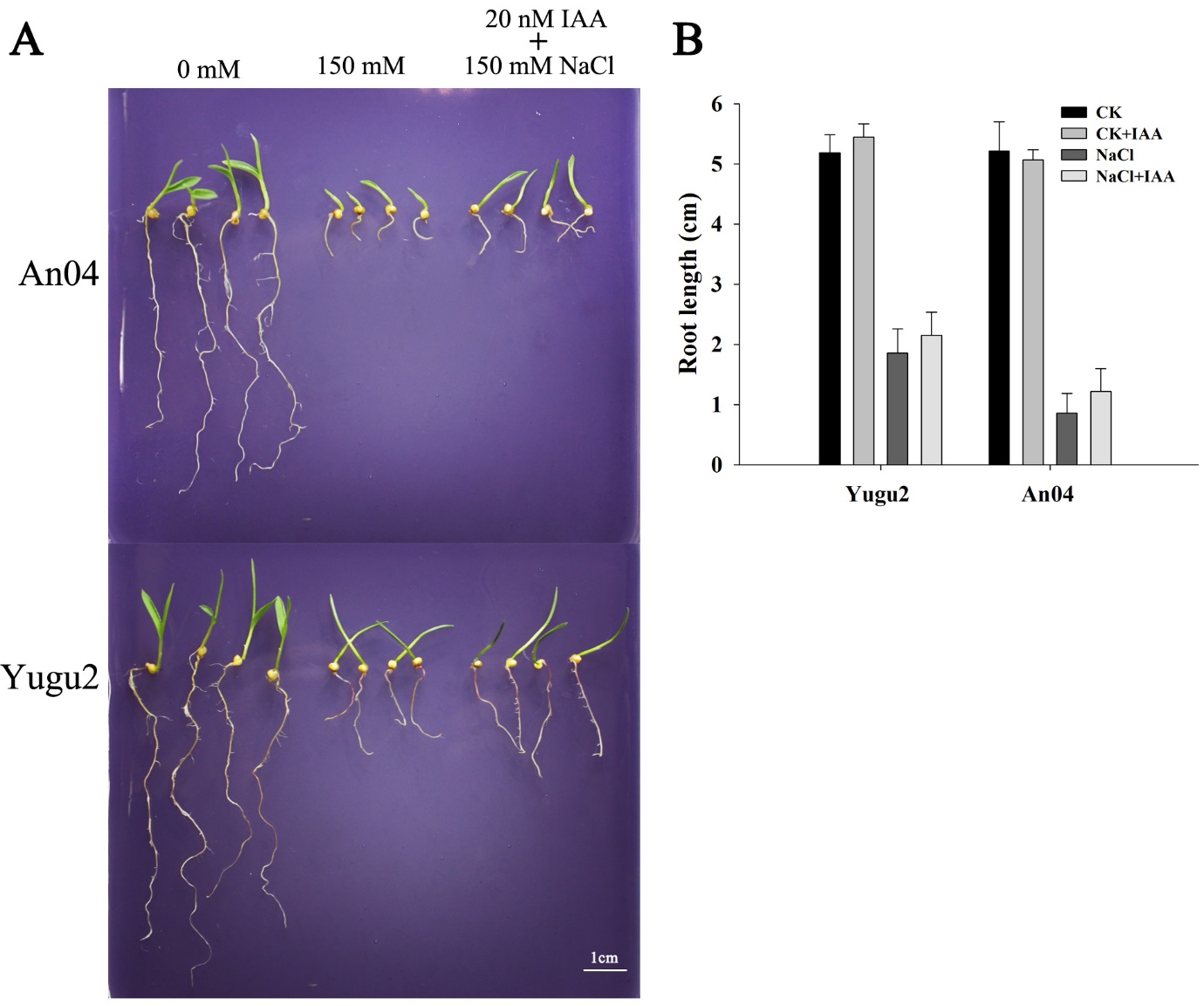

Supplement: Supplementary file 24 — Supplementary Figure S7. [file 41598_2020_70520_MOESM24_ESM.docx]
